# Mito Hacker: a set of tools to enable high-throughput analysis of mitochondrial network morphology

**DOI:** 10.1038/s41598-020-75899-5

**Published:** 2020-11-03

**Authors:** Ali Rohani, Jennifer A. Kashatus, Dane T. Sessions, Salma Sharmin, David F. Kashatus

**Affiliations:** 1 Department of Microbiology, Immunology and Cancer Biology, University of Virginia School of Medicine, Charlottesville, VA 22908 USA; 2 UVA Cancer Center, University of Virginia School of Medicine, Charlottesville, VA 22908 USA

**Keywords:** Computational platforms and environments, Image processing, Software, Mitochondria

## Abstract

Mitochondria are highly dynamic organelles that can exhibit a wide range of morphologies. Mitochondrial morphology can differ significantly across cell types, reflecting different physiological needs, but can also change rapidly in response to stress or the activation of signaling pathways. Understanding both the cause and consequences of these morphological changes is critical to fully understanding how mitochondrial function contributes to both normal and pathological physiology. However, while robust and quantitative analysis of mitochondrial morphology has become increasingly accessible, there is a need for new tools to generate and analyze large data sets of mitochondrial images in high throughput. The generation of such datasets is critical to fully benefit from rapidly evolving methods in data science, such as neural networks, that have shown tremendous value in extracting novel biological insights and generating new hypotheses. Here we describe a set of three computational tools, *Cell Catcher*, *Mito Catcher* and *MiA*, that we have developed to extract extensive mitochondrial network data on a single-cell level from multi-cell fluorescence images. *Cell Catcher* automatically separates and isolates individual cells from multi-cell images; *Mito Catcher* uses the statistical distribution of pixel intensities across the mitochondrial network to detect and remove background noise from the cell and segment the mitochondrial network; *MiA* uses the binarized mitochondrial network to perform more than 100 mitochondria-level and cell-level morphometric measurements. To validate the utility of this set of tools, we generated a database of morphological features for 630 individual cells that encode 0, 1 or 2 alleles of the mitochondrial fission GTPase Drp1 and demonstrate that these mitochondrial data could be used to predict Drp1 genotype with 87% accuracy. Together, this suite of tools enables the high-throughput and automated collection of detailed and quantitative mitochondrial structural information at a single-cell level. Furthermore, the data generated with these tools, when combined with advanced data science approaches, can be used to generate novel biological insights.

## Introduction

Mitochondria are important organelles that play multiple roles within the cell, including the regulation of oxidative metabolism, control of programmed cell death, calcium buffering and the generation of a variety of signaling metabolites^1^. Work over the past several decades has shown that mitochondrial shape significantly influences these various functions and cells can rapidly change mitochondrial shape to adapt to environmental conditions^2^. Furthermore, disruption of mitochondrial shape is associated with an increasing number of diseases, including cancer, diabetes and neurodegeneration^3^. Increasing interest in mitochondrial dynamics has been accompanied by a wealth of new reagents and tools to study mitochondrial structure and function. As the quality and sophistication of these tools has advanced, it has opened the door to creating large sets of mitochondria morphology data and applying advanced data science and machine learning approaches in order to generate novel biological insights^4–7^. The currently available tools offer a variety of different methods to classify and quantify mitochondrial morphology, ranging from the analysis of submitochondrial network structure, to analysis of mitochondria-level and cellular-level structure. When applied over large enough sets of images, many of these tools are capable of generating the types of data sets that can enable data science approaches. However, to fully realize this promise requires high throughput, fully automated, end-to-end mitochondria specific image processing and image analysis capabilities, that enable bulk analysis of images. For this matter, we sought to develop an unbiased set of methods to segment and analyze fluorescently labeled mitochondria from multi-cell images at a single cell level. In this article, we introduce Mito Hacker, which is a group of independent tools for high-throughput analysis of mitochondrial networks.

In order to generate single-cell data sets of mitochondrial morphology sufficiently large enough to enable machine learning (ML) applications, it is essential that individual cell identification, isolation, segmentation and quantification steps are fully automated.

**Figure 1:**
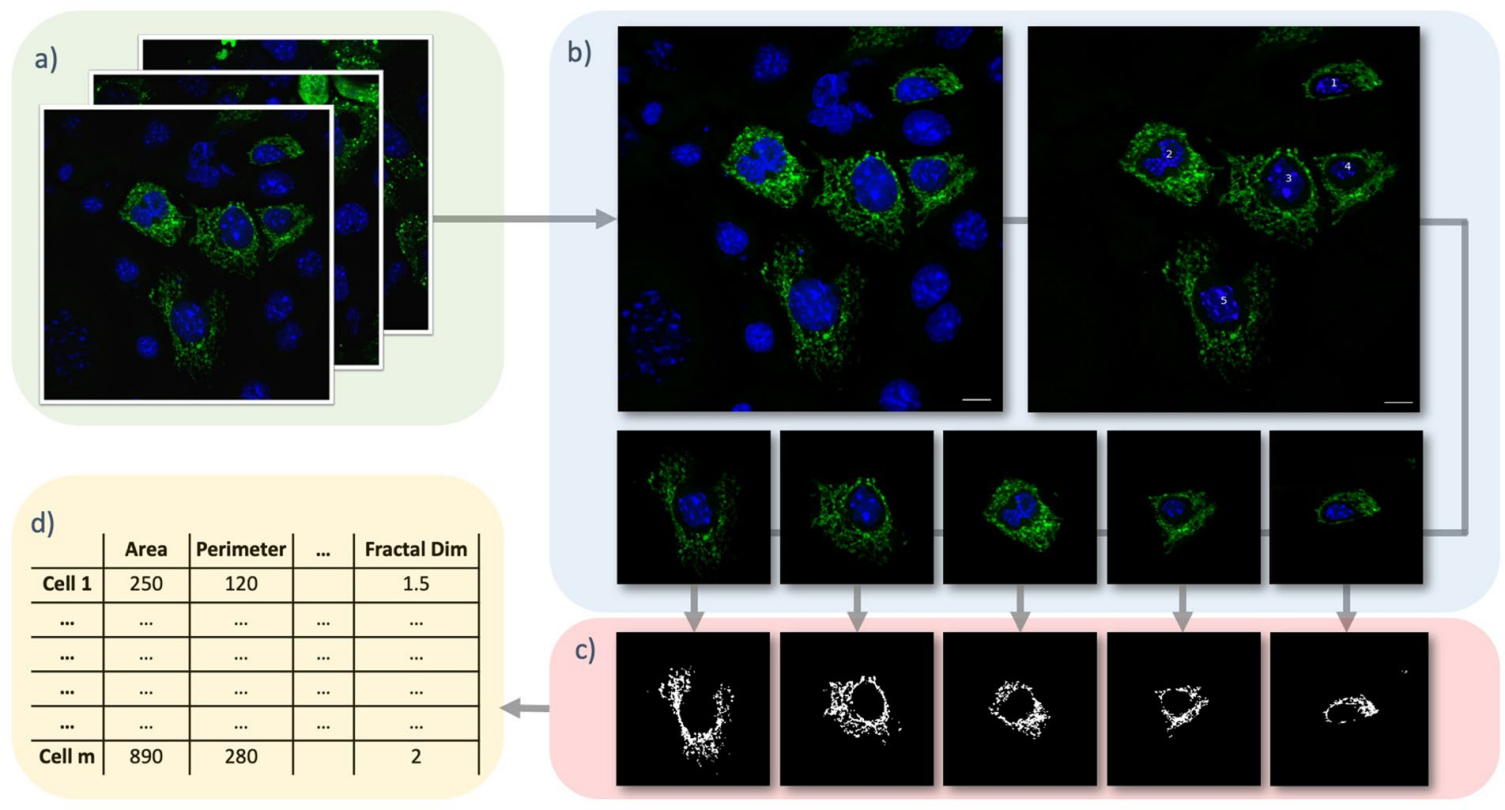
Mito Hacker Workflow. (**a**) Batch Analysis: Multiple images can be uploaded at the same time. (**b**) Cell Catcher: First, the ghost cells are identified and removed from each image, and then individual cells are separated based on Expectation Maximization (EM). (**c**) Mito Catcher: Pixel intensity distribution within the nuclear zone is used to estimate the background and signal levels to effectively segment the mitochondrial network. (**d**) MiA: The segmented mitochondrial networks are quantified using MiA, and the data for the quantified networks is exported in a tabular format. Scale Bars: 10 μm.

As such, we set out to develop a set of computational tools, Mito Hacker, to perform these key tasks on 2 dimensional multi-cell images with fluorescently labeled mitochondria. Mito Hacker is operating system agnostic and performs serial analysis of images on CPU. It offers a semi graphical user interface through Jupyter notebooks, and it is optimized to analyze 2D images of adherent tissue culture cells with mitochondria stained in the green or red channel and nuclei stained in the blue channel. Because Mito Hacker is designed to enable analysis of images generated in high throughput, we chose to optimize these tools for 2D, rather than 3D images. While 2D representations of mitochondrial networks that exist in 3D space can sacrifice some information on network connectivity, this sacrifice enables the collection and processing of the large number of images required for most advanced data science approaches. Mito Hacker has three major parts (Fig. 1): *Cell Catcher*, *Mito Catcher* and *MiA,* described below.

*Cell Catcher* is a tool designed to automatically identify, separate and isolate individual cells from 2D multi-cell RGB images (Fig. 1b). This tool uses the statistical distribution of mitochondria and nuclei across an image to separate individual cells and export them as single-cell images. Subsequently, these exported images can be used by the next tool, *Mito Catcher*, to segment the mitochondrial networks, or can be independently used to train various machine learning (ML) or neural networks (NN), such as Convolutional Neural Nets (CNN).

*Mito Catcher* uses the statistical distribution of pixel intensities across the mitochondrial network to detect and remove background noise from the cell and segment the mitochondrial network (Fig. 1c). Additionally, this tool can further improve the accuracy of the mitochondrial network segmentation through an optional adaptive correction, which takes the variation in the efficiency of fluorescence staining across each cell into account to enhance mitochondrial segmentation. Segmented mitochondrial networks are exported in binary and color formats, each of which can be used to train independent ML or NN models.

*MiA* uses the binarized mitochondrial network generated by *Mito Catcher* to perform greater than 100 mitochondria-level and cell-level morphometric measurements (Fig. 1d). *MiA* then exports the results as tabular data (CSV and TSV formats) for further analysis. The exported results include both raw and processed data to provide the user with maximum flexibility.

In order to offer flexibility and to make the tools applicable on a wide range of images, these tools have tunable parameters set by the user to extract the most accurate mitochondrial morphology data from their images. When applied consistently across experimental groups, these parameters can allow users to analyze images that span a broad range of quality while maintaining robust experimental design. A detailed description of these tunable parameters can be found in the Supplementary File (Aside I: Mito Hacker’s various functions and their parameters).

## Description of tools

### Isolation of single cells: *Cell Catcher*

Mitochondrial structure and function can be heterogeneous both within and between populations of cells^1^. This heterogeneity reflects the adaptability of the mitochondrial network and can contribute to both normal and pathological cellular physiology^3^. In order to properly analyze mitochondrial structure in populations of cells and to fully elucidate the importance of this structural heterogeneity, it is important to generate morphology data at a single cell level. While manual identification of individual cells is feasible for tens and potentially even hundreds of cells, advanced data science approaches require the collection of data from thousands to millions of cells. As a first step towards high-throughput analysis of mitochondrial morphology, we developed Cell Catcher, an automated cell separation method to detect and separate individual cells from multi-cell images.

Cell Catcher is designed to function on multi-cell images in which both nuclei and mitochondria are fluorescently labeled and it works based on the following assumptions: (1) Each mitochondrion on the image is uniquely linked to a single nucleus on the image. (2) Nuclei are stained in blue and mitochondria are labeled either red or green. (3) There are no in-plane mitochondria within the boundary of the nucleus. (4) All nuclei will be stained but there may be cells that lack identifiable mitochondria (ghost cells—e.g. untransfected cells in experiments using transient transfection of a mitochondrially-targeted fluorescent protein). Note that while Cell Catcher requires nuclear staining in addition to mitochondrial staining, to extend its application to cells without a nuclear stain, Mito Hacker offers an optional tool, Nuc Adder, in which the user can add synthetic nuclei to the images with a mouse click to make them compatible.

#### Ghost-cell removal

Since every mitochondrion is uniquely linked to a single nucleus (cell) in the image, our first step is to identify and remove ghost cells (i.e.—cells without detectable mitochondrial staining) to ensure they are not assigned mitochondria from other cells due to proximity. Ghost cell removal is a multi-step process, as described below.

#### Nuclear channel processing

We start by applying a top-hat filter to reduce the effects of the possible non-uniform illumination in the image^8,9^. Then we use a Gaussian blurring filter to decrease the granularity on the nuclei to have more uniform nuclear structures. Next, we create the nuclei binary mask. To achieve this, we use the distribution of the non-zero pixel intensities in the nuclear channel to calculate the binarizing threshold. Using dynamic thresholding based on the distribution of pixel intensities in the image, instead of using a pre-determined value for binarizing, results in more robust results in high throughput analysis of images. This method is especially effective when analyzing a batch of images that have variable signal intensities due to varying staining efficiency or different export settings. Once the nuclear mask is created, we consecutively apply morphological opening and closing filters to the mask to remove noise and fill holes in the nuclear structures. Next, we calculate the contours of the objects on the nuclei mask and fill them to ensure that our mask maximally covers the real nuclear area. Finally, we use a size filter to remove non-specifically stained objects from the nuclear channel.

#### Mitochondria channel processing

One of our assumptions is that there are no in-plane mitochondria within the nuclear zone. Thus, once we create the nuclear mask, we apply it to the mitochondrial channel and find the distribution of pixel intensities in the masked area to estimate the overall background intensity in the mitochondrial channel. By approximating the pixel intensity distribution with a normal distribution, we define the threshold for the signal level at the mitochondrial channel as ($mean + 2 \times standard\;\; deviation$) which equates to a 95% probability of being a mitochondrial signal rather than background noise. Then, we use this calculated threshold to globally remove background noise from the mitochondrial channel.

#### Identifying ghost cells

After removing the background noise, we assume the remaining pixels in the mitochondrial channel represent mitochondria. Thus, we can measure the amount of mitochondrial content within a certain distance from each nucleus by simply adding the pixel intensities together within that region. If the mitochondrial content in the neighborhood of a particular nucleus is below a certain threshold (set by the user), we flag that nucleus (cell) as a ghost and remove it from the nuclear channel. Identifying and removing ghost cells helps us to avoid false mitochondrial linking to these unstained cells and improves cell separation accuracy and speed.

#### Mitochondria-cell linking

**Figure 2:**
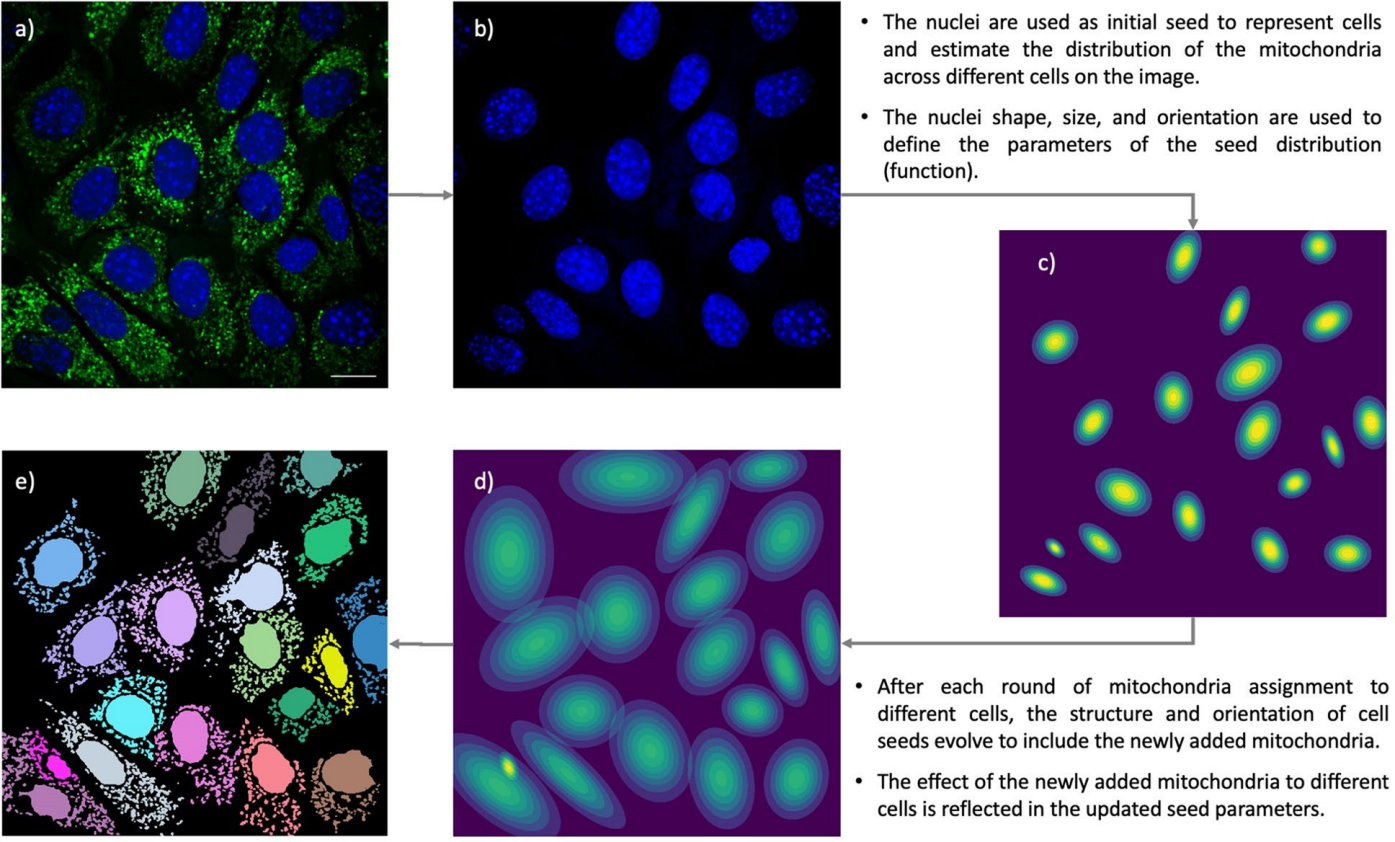
Cell Catcher isolates individual cells on each image using the expectation maximization. (**a**) A multi-cell image uploaded to Cell Catcher. (**b**) Nuclei are segmented and separated. (**c**) The nuclei are used as initial seed to estimate the distribution of the mitochondria across different cells on the image. (**d**) After various rounds of mitochondria assignment, the seeds’ structures and orientation are updated to reflect the effects of newly added mitochondria at each step. (**e**) The final map of individual cells on the images after separation by Cell Catcher. Scale Bars: 10 μm.

After removing the ghost cells, we assign mitochondria to cells in a multi-step process. The mitochondrial assignment is based on the Expectation Maximization (EM)^10^. The process begins with the nuclei identified as seeds for the cells in the image (Fig. 2a–c). Next, we measure the likelihood of each mitochondrion belonging to different nuclei and assign each mitochondrion to a cell with the maximum likelihood. For this matter, we consider any single object on the image that survived the background removal a mitochondrion. After the first round of assignments, we update the distribution of the cells and repeat the assignment process until there are no new assignments.

The first step in assigning mitochondria is to divide mitochondria into three mutually exclusive yet collectively exhaustive groups: (1) Small mitochondria that make direct contact with a particular nucleus; (2) large mitochondria that contact one or more nuclei; and (3) free-floating mitochondria with no obvious connection to a particular cell. The assignments for the first group of mitochondria are straightforward and we directly add them to the seed that they contact and update the seed distribution (Fig. 2c).

For the second group of mitochondria that contact multiple nuclei and thus cannot be immediately assigned, we assign this group of mitochondria to seeds pixel by pixel. For each pixel, we measure the likelihood that it belongs to the distribution derived from each seed in the image, and then assign it to the seed with the maximum likelihood (Fig. 2d).

Following the first two rounds of assignments and updated seeds, we next measure the average likelihood of each remaining mitochondrion (group three) belonging to each seed in the image. The entire mitochondrion is then assigned to the seed with the maximum likelihood (Fig. 2e).

#### Linking validation and cell correction

**Figure 3:**
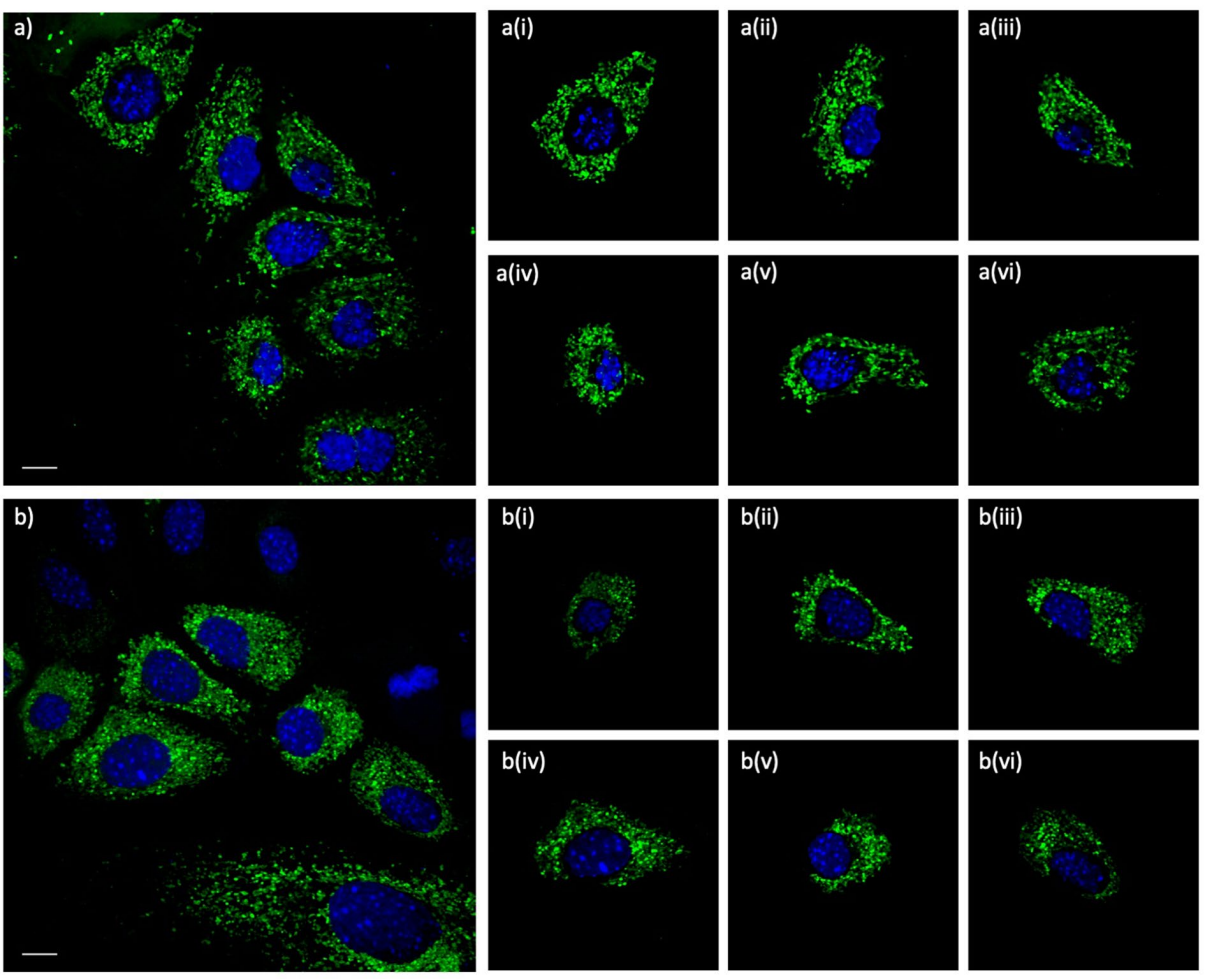
Single cell isolation using cell catcher. Murine pancreatic cancer cells were transiently transfected with mito-YFP and imaged using confocal microscopy. (**a**,**b**) Sample multi-cell images processed by Cell Catcher to identify and separate individual cells. The two cells on bottom right in (**a**) and (**b**) that have substantial overlap with the frame edges are identified as bad cells by Cell Catcher, automatically exported to a separate folder and excluded from subsequent analysis. Excluded images remain available to the user. Scale Bars: 10 μm.

Initially, we assumed that each mitochondrion in the image belongs to a cell in the same image, but this is not always a valid assumption. Often, mitochondria appearing on the edges of the frame belong to cells whose nuclei are not on the frame. We need to identify these mitochondria in order to remove their incorrect linkage to nuclei visible on the frame. After finishing the initial mitochondria-cell assignment, we use the 2d distribution of the centers of the mitochondria across each cell and apply the Mahalanobis' distance (threshold 5.99 (df = 2, p = 0.05)) to identify outlier mitochondria for each cell. Based on the Chi-square distribution, Mahalanobis' distance is a statistical measure of the distance that takes into account correlations in the data by incorporating the covariance of the data distribution in measuring distances (Supplementary Fig. 1). Following this step, each cell, with its assigned mitochondria, is exported as a single-cell image that can be used for further analysis (Fig. 3). Cell Catcher was developed using a variety of cell types, including murine pancreatic tumor cells transfected with mitochondrially-targeted YFP (Fig. 3) and Human Embryonic Kidney cells stained with MitoTracker Red (Supplementary Fig. 2). We have also validated that Cell Catcher can function on two independent sets of publicly available images from the Allen Institute for Cell Science (AICS—http://www.allencell.org) and from the Broad Bioimage Benchmark Collection (data.broadinstitute.org/bbbc/index.html) (Supplementary Figs. 3–5), though its performance decreases as cell density approaches 100% confluence.

### Cell segmentation: *Mito Catcher*

#### Pre-processing

To enhance the accuracy in quantifying mitochondrial network morphology, we require sharp and low noise images. At this point, we have already adequately processed our nuclei during the cell separation step (Cell Catcher). However, we still need to prepare the mitochondrial network. To do this, we first apply a top-hat filter to the mitochondrial channel to reduce noise and increase sharpness. This step may be followed by application of Contrast Limited Adaptive Histogram Equalization (CLAHE)^11^, which adjusts the pixel intensities to equalize the histogram throughout the image. While this step is useful in enhancing image contrast, it may introduce an excessive amount of noise in certain sets of images and result in unfavorable outcomes. Thus we have left it as an optional step. Finally, a median filter is applied to remove the inherent salt and pepper noise in the image, which can also be amplified by application of the previous two filters.

#### Thresholding

Due to the inherent nonuniformity of fluorescence signal distribution across mitochondria, finding a proper threshold to accurately binarize the mitochondrial network can be difficult. While a threshold that is too high may result in loss of mitochondria, a too low threshold may create connections that don't exist and result in over-representation of the network. Threshold selection is an even bigger challenge in high-throughput analysis of multiple images since manually setting a threshold for each individual image is generally not feasible. To solve this problem, we developed a method that uses the statistics of the fluorescence signal distribution in each cell to identify the proper binarizing threshold.

We have identified the nuclear area as an appropriate zone to measure the background signal in the mitochondrial channel. First, this area is definitely within the boundaries of the cell, and second, the mitochondrial fluorescence signal in this area represents the out-of-plane signal, which will also be present across the whole cell. Thus, by measuring the signal level in this area, we are effectively approximating the background level throughout the whole cell and can use it to threshold the mitochondrial network. After measuring the pixel intensities in this area, we find the first and third quartiles (${\text{Q}}_{1}$, ${\text{Q}}_{3}$) along with the interquartile range (IQR) of the pixel intensity distribution. Then, by keeping the pixel intensities within a range defined by (${\text{Q}}_{1}-1.5\times \text{IQR}, {\text{Q}}_{3}+1.5\times \text{IQR}$), we identify and remove the outlier pixel intensities from our background signal estimation.

After removing outliers, we calculate the mean and the standard deviation of the remaining pixel intensities (which represent real background pixels) in this region. We assume that pixel intensity distribution follows a normal distribution. By using the properties of the normal distribution, any pixel with an intensity higher than the mean + 2 standard deviations is only 2.5% likely to be a background signal. Thus, we use this value as the signal threshold to separate the mitochondrial network from the background^12^.

#### AdaMM—Adaptive Mitochondrial Masking

**Figure 4:**
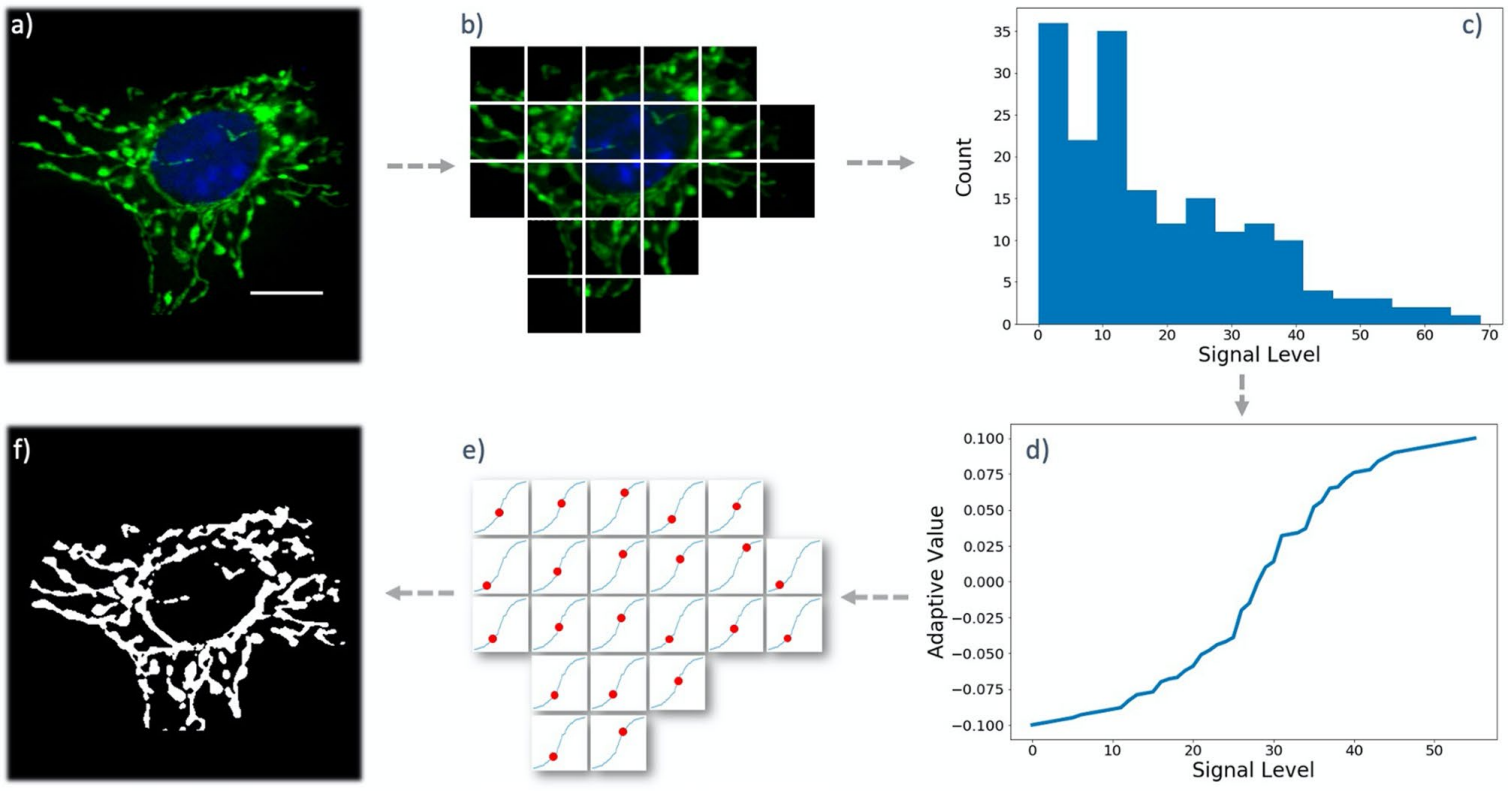
Adaptive Mitochondrial Masking (AdaMM). (**a**) Image of a sample cell to be analyzed. (**b**) The image is divided into equally sized blocks, and the empty blocks are removed. (**c**) The distribution of average signal across the tiles is calculated. (**d**) A generalized logistic function is fit to the tile intensity distribution to determine the level of correction needed for each tile based on its signal level. (**e**) Each tile receives a different correction factor, and the adjusted threshold is used to locally segment the contents of each tile. (**f**) The segmented tiles are attached together to create the final, locally thresholded image of the cell for analysis. Scale Bars: 10 μm.

While the previously described mitochondrial thresholding method has been sufficient to segment mitochondrial networks in a number of test experiments, in certain cases we were able to further enhance mitochondrial network quality through an adaptive local threshold correction (Fig. 4). To achieve this, we break down the image of the cell into equally-sized tiles and measure the average pixel intensity in each tile (Fig. 4b). We then compare these intensities with the average pixel intensity in the nuclear zone, which we use to calculate the global segmentation threshold.

On the one hand, applying a lower segmentation threshold on the tiles that have weaker signal levels compared to the background signal retains regions of the mitochondrial network that would otherwise partially or fully lose structural definition under a stronger global threshold. On the other hand, raising the segmentation threshold over the tiles that have higher signal levels helps to remove non-existing connections within the segmented mitochondrial network.

To achieve adaptive threshold correction, we initially find the distribution of the average signal intensity among all the tiles in the image (Fig. 4c). Next, we fit a generalized logistic function with an inflection point on the average background intensity (measured within the nuclear zone) to this distribution (Fig. 4d). By default, the tiles with average signal levels equal to the background mean will not be corrected for threshold. However, the remaining tiles will either experience a boost or drop in their threshold levels (Fig. 4e). The degree of the threshold correction in each tile is determined by the growth rate, horizontal asymptotes, and the offset of the fit, which can be set by the user.

The ultimate output of Mito Catcher, following these pre-processing and thresholding steps, is a segmented mitochondrial network that is exported in binary and color formats, each of which can be used to train independent ML or NN models. In order to validate the quality of the automated segmentation, we set out to compare the output of Mito Catcher to hand segmentation. To do this, we assigned 5 cells to two different users and asked them to segment the mitochondria in those 5 cells by drawing over them using a computer mouse (or their touchpad). We also used Mito Catcher to segment the individual cells. We then compared results from the users with each other and with the segmentation results from Mito Catcher (Supplementary Fig. 6).

First, we created a binary mask from the user drawings to be the mitochondrial segmentation mask in each cell. To compare each pair of images (Mito Catcher vs. user1; Mito Catcher vs. user2; and user1 vs. user2), we overlaid the binary masks and generated two masks representing their overlapping areas: common between the two masks (logical AND) and their union (logical OR). Using these masks we calculated three different parameters to compare different segmentation results:

##### The effective overlap

This parameter is calculated by dividing the intersection to the union of the masks generated by two different sources. This measure is robust to selecting extreme settings for Mito Catcher. For instance, if we use a set of settings that results in a fully connected network in a cell, it would result in a denser mask, and consequently it would increase the intersection area with the user generated mask. However, this action would also increase the union of the two masks (the denominator), and consequently the effective overlap would be a robust measure of similarity.

##### The percentage of the mitochondrial mask captured by another source

This parameter calculates the percentage of the user defined mitochondrial mask that is captured by Mito Catcher or another user.

##### The ratio of captured mitochondrial content by different sources

This measure compares the ratio of the mitochondrial content (mitochondrial mask area) marked by two different sources. The goal for this measure is to compare if Mito Catcher is either catching too much noise as mitochondria, or it is missing too many mitochondria in general. This feature is also robust to selection of extreme settings in Mito Catcher.

Comparing the segmentation results by different users on the same image shows that for several reasons, the absolute value of the measured features is not useful in practice and their relative value is more informative. First, different users often differ whether an object on the image is a mitochondrion or not. This occurs more often in images with lower quality or lower signal to noise ratio (SNR). Second, using a computer mouse or touchpad to draw over mitochondria is prone to error. In some cases, the drawn lines are thinner or thicker than the original mitochondria, or the lines are slightly off-center (although still covering the mitochondria).

Due to these errors, the average effective overlap between the user segmented mitochondria among different cells was 65%, which suggests that there is no single gold standard segmentation in cells with average quality and average SNR (the majority of images in typical experiments). Consequently, any absolute measurement would be biased and prone to error.

Supplementary Figure 6 shows the detailed results for one of the tested cells, along with average results for the 5 tested images. We use the generated masks by the two users, and the mask generated by Mito Catcher, to measure the average relative performance of Mito Catcher in terms of the above parameters: ((Mito Catcher vs. User 1 + Mito Catcher vs. User 2)/(User 1 vs. User 2)). The average effective overlap between the Mito Catcher generated mask and the user generated masks is 96.5%, the average percentage of the mitochondrial mask captured by Mito Catcher compared to different users is 94.3%, and finally, while the amount of segmented mitochondrial content is 9% different among different users, the average amount of mitochondrial content segmented by Mito Catcher is about 1% different than user segmented mitochondria. All of these measurements suggest that Mito Catcher segmentation efficiency is comparable to manual segmentation.

As further validation of the quality of Mito Catcher segmentation, we ran Mito Catcher on the set of single-cell images generated by Cell Catcher using the publicly available data set from the Allen Institute for Cell Science (Supplementary Fig. 4). The visible mitochondrial detail in these binary single-cell images is comparable to the segmentation provided with this publicly available data set (Supplementary Fig. 7, compare to Supplementary Fig. 3).

### Morphological measurements: MiA

Scientists have adopted different approaches to quantify mitochondrial morphology in cells^8,13–21^. A closer analysis of the developed methodologies reveals that they lie within two major categories^22^: (i) Morphological classification of mitochondria. The tools in this category attempt to identify different mitochondrial morphologies and measure the properties of each morphological subset as the basis for comparison^20,23–25^. (ii) Morphometric measurements of mitochondria. There are a wide variety of tools available in this category. Based on the scope, and level at which these tools perform mitochondrial measurements, these tools form three main sub-categories: (a) Mitochondrial level measurements: the methodologies in this sub category treat each mitochondria as an individual entity, and perform various morphometric and geometric measurements, such as area, length, width, aspect ratio, compactness, form factor^8,26–31^; (b) Sub-mitochondrial level measurements: these tools aim to quantify the underlying mitochondrial network structure in the cell. These tools extract the skeleton of the mitochondrial network, find mitochondrial junctions and endpoints, and measure properties such as branch length and number of branches in individual mitochondria and across the whole mitochondrial network of the cell^8,13,29,32,33^; (c) Cell level measurements: there are no tools that solely perform cell level measurements. The tools in this sub-category, originally belong to the other sub-categories of the morphometric measurements. However, by aggregating their respective measurements over the population of mitochondria within the cell and expanding their scope, they form a new sub-category of morphometric measurements^29,33^. It is worth mentioning, while most of the tools in each category are focused on a particular set of relevant measurements, some of the tools have a larger scope and go beyond a single category or sub category. For instance, Mytoe^8^ and Nikolasiensen et al.^29^ offer mitochondrial and sub-mitochondrial level measurements, and Leonard et al.^14^ offer Morphological classification, and mitochondrial level morphometric measurements. While the majority of the available tools perform morphometric measurements on 2D images, some tools like MitoGraph^33^, MitoMap^31^ and Nikolasiensen et al.^29^ extend morphometric measurements to the third dimension.

The current version of MiA, along with the rest of the tools available in Mito Hacker, offers 2D mitochondrial network analysis, and fits into the Morphometric measurements category. As discussed earlier, all the available tools within this category treat individual mitochondria as an independent entity and some of them generate the cell level measurements by aggregating the readouts from each mitochondrion. MiA collects the majority of these mitochondria-level and sub mitochondrial-level measurements (~ 90% of the cumulative number of features offered by the tools studied earlier). However, MiA uniquely complements these measurements by a set of direct cell-level measurements that take the distribution of mitochondria across the cell into account. This qualifies MiA as the only tool that independently lies within the all the three groups of the morphometric measurements’ category.

#### Mitochondria-level measurements

**Figure 5:**
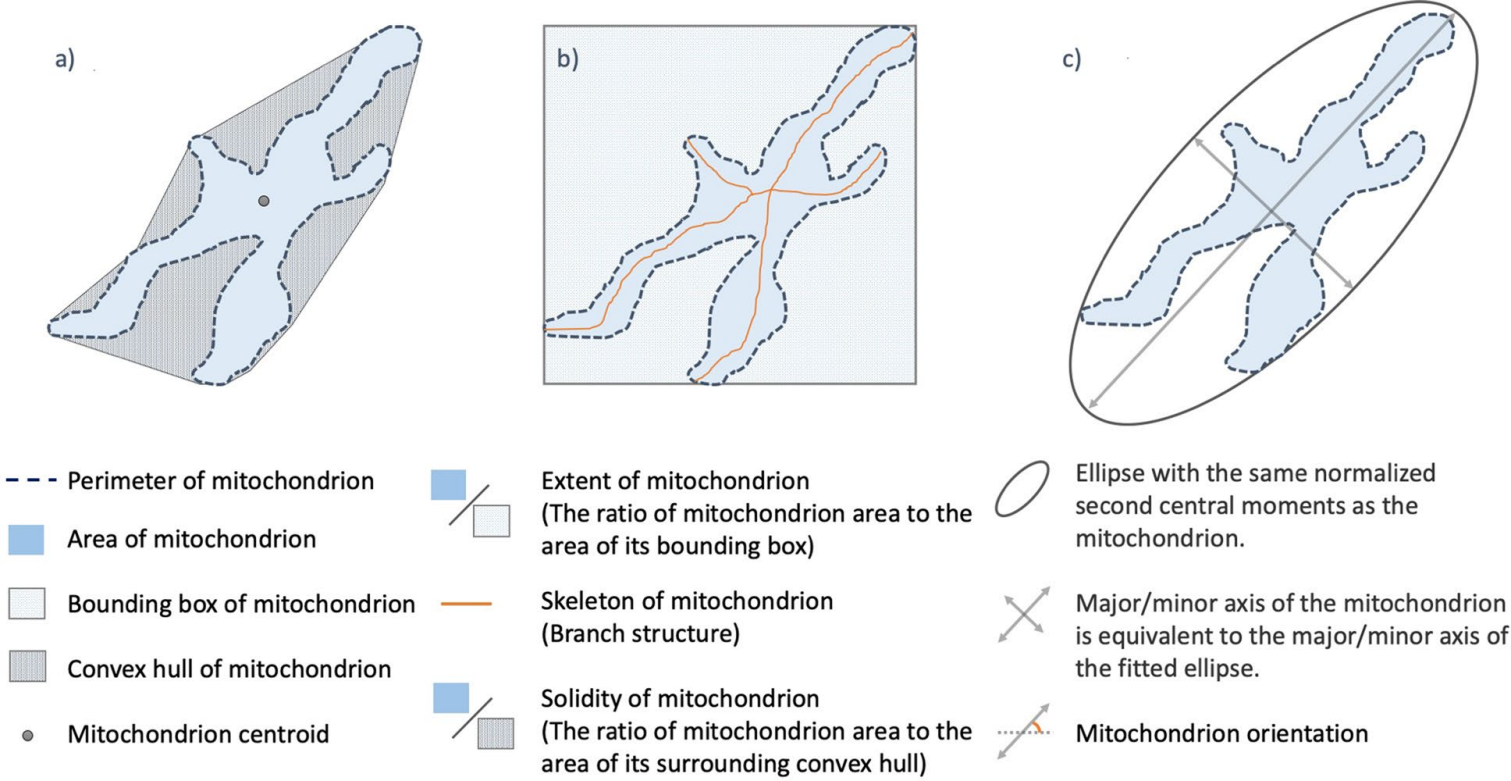
Morphological measurements from MiA. (**a**) A schematic of a mitochondrion showing the basic geometrical measurements such as area, perimeter, convex hull and the solidity of mitochondrion. (**b**) Measuring the bounding box of a mitochondrion and its extent. The orange lines depict the skeleton of the mitochondrion, which is extracted through a thinning process. (**c**) Measuring the major and minor axes of mitochondria and its orientation.

MiA measures thirty-one distinct morphological properties from each mitochondrion (Fig. 5, Supplementary Table 1). Twenty-three of these measurements directly quantify the geometry of mitochondria, by measuring properties such as area, perimeter, roundness, form factor, etc. The remaining eight measurements are aggregations of branch-level properties such as total, mean, and median length of the individual branches that make up each mitochondrion. To perform these branch-level measurements, we first extract the skeleton of each mitochondrion through a thinning process. During thinning, we continuously erode the mitochondrion from outside to a point where further erosion results in loss of connections in the mitochondrial structure. The remaining structure is the wireframe of the mitochondrion and individual branch points can be defined as the intersection of three or more wires. The lengths of the skeleton between each branch point are defined as individual branches.

#### Cell-level measurements

We measure 110 distinct features from each cell. Ninety of these features are different aggregations of the mitochondria-level measurements such as mean, median, and the standard deviation of the area or perimeter of all mitochondria in the cell. The remaining twenty features are measurements of the entire population of mitochondria and their distribution across the cell. For instance, mitochondrial network solidity is the ratio of mitochondrial network area to the area of the convex hull enclosing the network (See Supplementary Table 1). This feature represents the degree of sparseness in the distribution of mitochondria across the cell. Another example is the fractal dimension of the mitochondrial network^21^. This property estimates the geometrical complexity, and the irregularity of shapes and patterns observed in the mitochondrial network in each cell. Full descriptions of the mitochondrial measurements performed by MiA can be found in Fig. 5 and Supplementary Table 1.

### Validation of the use of Mito Hacker for high-throughput data analysis

**Figure 6:**
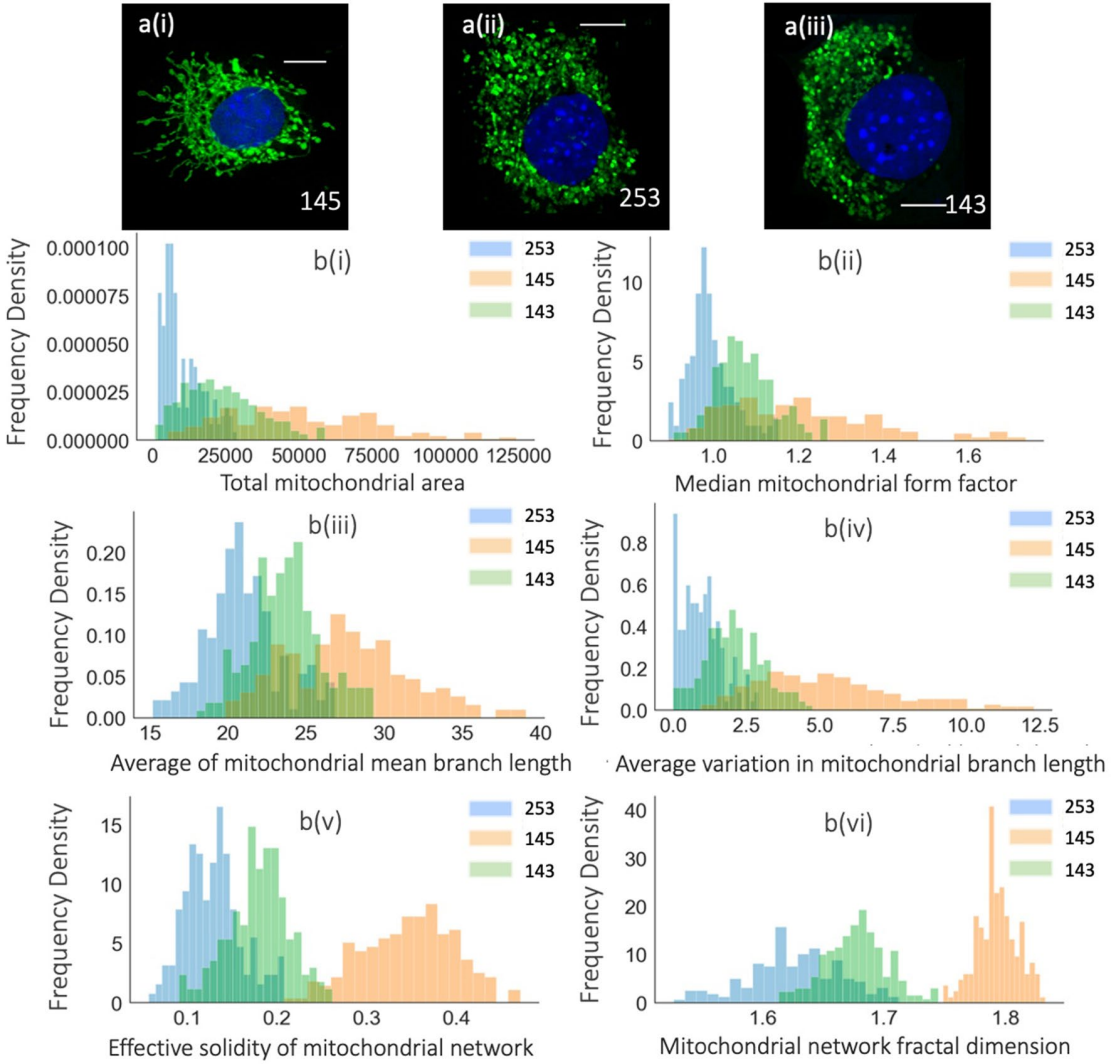
Analysis of Mito Hacker-generated data. (**a**) Representative images of KPDC145 (Drp1^−/−^), KPDC253 (Drp1^+/+^) and KPDC143 (Drp1^+/−^). Qualitative analysis of mitochondrial morphology indicates that KDPC145 is morphologically distinct from KPDC253 and KPDC143. (**b**) Distributions of various mitochondrial measurements from > 185 individual cells per cell line; (i, ii) Morphological measurements at the mitochondria level; (iii, iv) Morphological measurements at the sub-mitochondria level (mitochondrial skeleton); (v, vi) Morphological measurements at the cell level. Scale Bars: 10 μm.

The rationale for the development of Mito Hacker was that large data sets of mitochondrial morphology would enable scientists to identify novel relationships between mitochondrial morphology and cellular physiology. For example, this type of analysis across multiple heterogeneous cell lines, when combined with other “omics” approaches might lead to the identification of novel regulators of mitochondrial shape or physiological consequences of shifts in mitochondrial morphology. As a test of this approach, we took advantage of a set of murine pancreatic tumor cell lines we had generated that differ in their expression of the mitochondrial fission GTPase Drp1. We used Mito Hacker to generate a database of morphological features for 630 different cells from three different cell lines (KPDC145, KPDC143 and KPDC253) that encode 0, 1 or 2 copies of the Drp1 gene respectively and exhibit a range of mitochondrial morphologies^34^. We then employed Mito Hacker to quantify mitochondrial morphology from between 185 and 228 cells of each cell line (KPDC145: 228, KPDC143: 217 and KPDC253: 185) and exported the results in CSV format (Fig. 6 and Supplementary Figs. 8–10).

Since Mito Hacker validates each data point at the time it is generated, our final dataset is clean and has no missing or infinite values, which saves a large amount of effort and time during the analysis process. However, before building any models, we aim to identify the potential outliers in our data. To find outliers, we looped over the set of features and used the IQR outlier detection to decide whether the readout for each cell in that particular feature is potentially an outlier. Then we counted the number of outlier readouts for each biological cell (1 row) and tagged it as an outlier if more than 20% of its readouts were outliers.

After detecting the potential outlier cells, we inspected the original images and the mitochondrial mask for each outlier cell. This process revealed that the majority of these flagged cells either suffered from non-uniform staining or excessive external noise, which our binarizing method could not efficiently handle. After inspecting and removing potential outliers, we lost about 5% of the original cells, which we considered reasonable considering the quality of the discarded cells.

#### Data modeling

Once we had a clean set of data from the three cell lines, we set out to develop a predictive model that could use the mitochondrial morphology data to identify the cell line from which the data came. The rationale for this exercise is that if Mito Hacker is generating robust data on mitochondrial morphology, we should be able to build a model in which Mito Hacker-generated mitochondrial morphology data can predict whether a cell line has 0, 1 or 2 alleles of the mitochondrial fission regulator Drp1. We used Pandas^11^, Numpy^35^, Scipy^36^, Sklearn^37^, and XGBoost^38^ libraries to carry out the data quantification and modeling. As mentioned earlier, the majority of the features measured by Mito Hacker are different aggregations (mean, median, standard deviation) of mitochondrial morphometric measurements, which is expected to cause strong multicollinearity among features. While this multicollinearity may degrade the performance in many linear models, tree-based models are inherently immune to this phenomenon.

The tree-based models make no assumptions about the relationships between different features and split the data based on the features that maximally reduce the impurity at each step. So, when a particular feature splits the data, a highly collinear feature will not considerably improve the information gain over the next split, and consequently, the model will ignore it.

**Figure 7:**
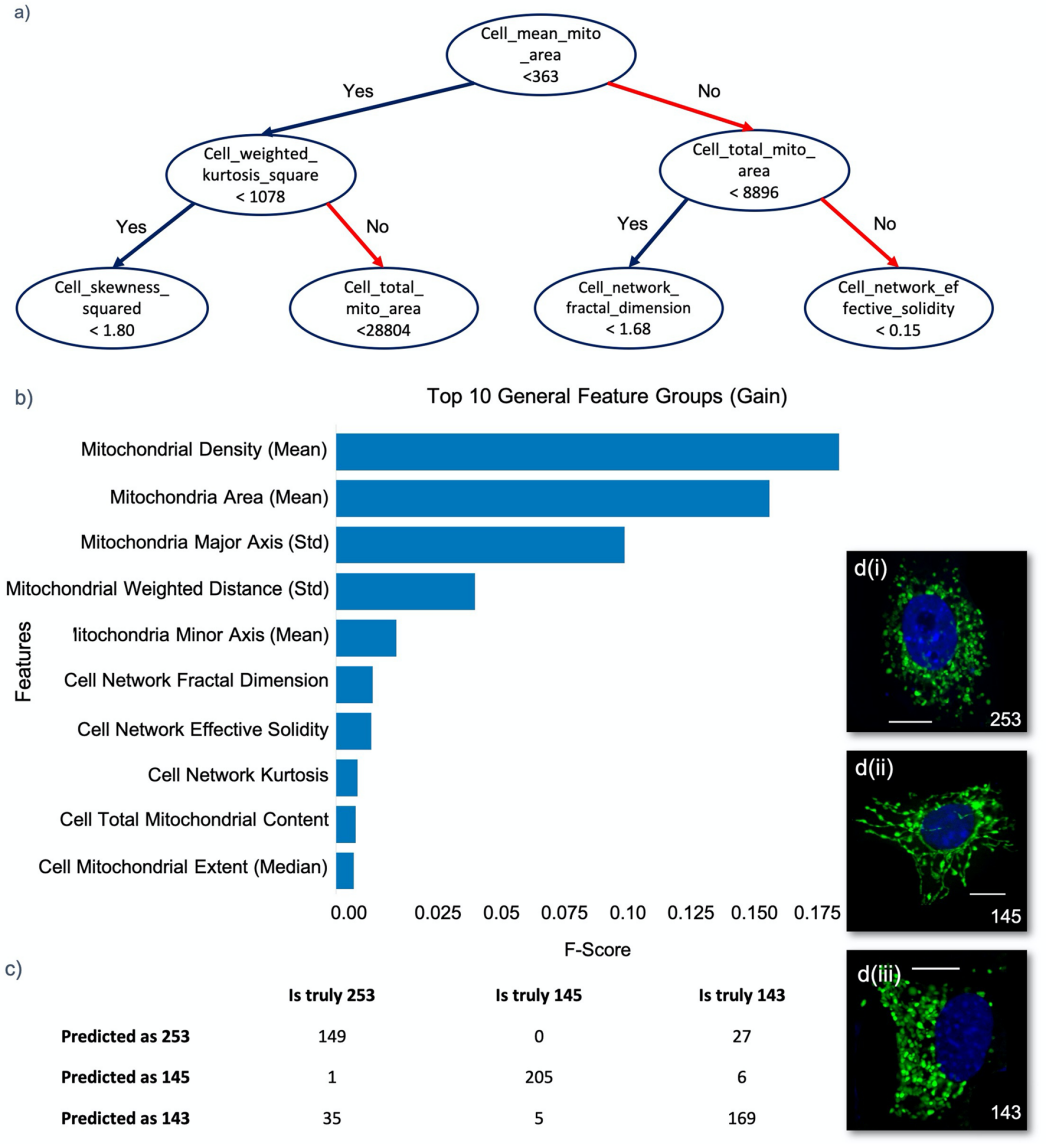
Predictive modeling using Mito Hacker-generated data. (**a**) The decision structure behind the classification of the three different cell lines. The structure suggests that the average of the area of the mitochondria across the cells is a strong predictor of the cell line. (**b**) List of the important features in distinguishing the morphological structures among different cell lines, ranked based on the achieved information gain after splitting the data. This is the ranking of the features after removing the highly correlated features from the dataset, using the IVF method. (**c**) The confusion matrix of the model after outlier removal. The matrix shows that the majority of the drop in the performance happens because the model is not able to effectively separate KPDC253 and KPDC143 (~ %75 accuracy). (**d**) Three representative cells from each of the three different cell lines demonstrating the apparent morphological similarity between KDPC253 and KPDC143. Scale Bars: 10 μm.

In addition, advanced tree-based models have shown promising performance in modeling various datasets. Interpretability is another advantage of the tree-based models. Decision structure and feature importance are two byproducts in tree-based models that can help to better understand the data and potentially extract biological meaning (Fig. 7). Due to these advantages, we chose a tree-based algorithm to model our data.

#### Model selection

Our ultimate goal for this type of analysis is to develop a set of models that (1) can use mitochondrial morphology to classify the cells based on a set of defining variables such as cell line identity, genotype, or some other relevant physiological readout, with high performance and accuracy (in this test case, we are predicting cell line identity); and (2) provide us with the feature importance list and the decision-making structure to help us better understand our data and extract biological insights. The tree-based models seem to be able to deliver both.

We trained different tree-based models (Decision Tree, Random Forest, AdaBoost, XGBoost) to classify the cells into their known cell lines (differing by Drp1 genotype). Each potential model was tested using a stratified tenfold cross-validation (CV) to ensure different cell lines are similarly represented in each fold.

Tenfold CV randomly divides the data into ten equal parts. One of the ten parts is held out as the validation set and is used to test the performance of the model. The remaining nine pieces are mixed and are used to train the model. After training, we evaluate the performance of the model by its ability to correctly predict the class of the cells in the hold-out sample (which we did not use for training). We then repeat this process nine more times (one time for each of the remaining validation sets). The average of the ten performance measures represents the performance of the model. By using a tenfold CV the data from each cell is used nine times for training and only once for testing. Thus, the entire dataset is used for both training and testing the model. We used the accuracy of predictions, which ranged from 60 to 87% among the tested models, to compare the different models. Among these, XGBoost achieved the highest accuracy in predicting the cell lines^38^.

As the name suggests, XGBoost is a boosting algorithm. The idea behind boosting is to employ a set of weak learners, each of which is slightly better than random guessing. By leaving the examples that the weak learners can handle and adding new weak learners with emphasis on handling the difficult observations, boosting methods achieve high accuracy in predictions.

XGBoost starts with a simple decision-tree as a weak learner. Then, one at a time, it adds additional trees to the model while existing trees in the model remain unaltered. This procedure minimizes the prediction loss sequentially by adding additional trees to the model.

To avoid overfitting, we controlled the complexity of the model by limiting the depth in each tree (6). Also, to make our model more robust, we applied a relatively small learning rate (0.1) to weigh the predictions of the newly added trees at each step.

To tune hyperparameters of our model, we started by using the default values provided by the XGBoost library in our first model. Then we used an exhaustive grid search with tenfold cross-validation to find the best combination of the hyperparameters, which yield the highest cell-line prediction accuracy.

Application of this XGBoost algorithm on our set of cells, using tenfold cross-validation, resulted in *87% average accuracy*, *87% average weighted precision* & *87% average weighted recall* in predicting the Drp1 genotype of the cell, where random selection would result in 33%.

$$Accuracy=\frac{TP+TN}{TP+TN+FP+FN} \quad Recall=\frac{TP}{TP+FN} \quad Precision=\frac{TP}{TP+FP}$$

where, TP = True Positive, TN = True Negative, FP = False Positive and FN = False Negative.

We found this high degree of accuracy to be important validation that the data generated by Mito Hacker is robust, especially given the apparent similarity of the mitochondrial staining from KDPC253 and KPDC143, which both express Drp1 (Figs. 6, 7).

#### Feature importance

The high degree of accuracy of this model confirms that Mito Hacker-derived data sets can be used to build highly accurate predictive models. However, the other goal of our analysis is to identify a feature importance list and to use the decision-making structure to help us better understand our data. Traditional approaches to identify regulators of mitochondrial morphology or to understand the consequences of shifts in mitochondrial morphology carry implicit assumptions about the set of mitochondrial features (e.g. length, aspect ratio) that are relevant. This inherent bias may ignore important features or focus too heavily on features with limited value. Our approach avoids this bias by performing a wide range of morphological measurements without making any assumptions about their importance and using the feature importance identified in our model to identify the most informative set of features in our data set. By adopting this approach, we are not limited to a fixed and pre-determined combination of features to study our cells, and we let the dynamics of the model discover the most relevant set of features in each study. However, as discussed earlier, there is strong multicollinearity between our features. This multicollinearity has minimal influence on performance in tree-based models, but it can artificially inflate the importance of collinear features. To mitigate this effect, we used the variance inflation factor (VIF) to identify and remove the highly inflated features from our data set. After removing the highly inflated features, we trained our model with the remaining features and achieved 80% accuracy in predicting the correct cell line despite removal of 60% of the redundant features. The resultant feature list (Fig. 7b) shows that we can identify key features that distinguish these three cell lines while maintaining a high degree of accuracy. Interestingly, analysis of the feature list reveals that in this predictive model, mean mitochondrial density and mean mitochondrial area contributed the most to distinguishing these three cell lines from each other. As mitochondrial area is a readout that reflects mitochondrial size, this is consistent with the known role of Drp1 in the regulation of mitochondrial fragmentation. Mean mitochondrial density, on the other hand, is the mean of the distances of the pixels in each mitochondrion from the boundary of the mitochondrion, which effectively measures the width of each mitochondrion. The importance of this feature may reflect other differences in shape that arise as a consequence of changes in Drp1 levels.

## Discussion

The use of large data sets and machine learning approaches is revolutionizing the process of biological discovery. For example, these approaches have been used to predict gene expression from epigenetic features and to predict the response of cancer cell lines to certain drugs^39^. Recent advances in high-content, high-throughput imaging have set the stage for this type of approach to be used to better understand the role of mitochondrial shape changes in cellular physiology. In this paper, we describe a set of tools that enables single-cell analysis of mitochondrial morphology using multi-cell fluorescence images. Furthermore, we have validated that the mitochondrial data generated by Mito Hacker are sufficient to build predictive models to distinguish genetically distinct cell lines.

As interest in mitochondrial dynamics has grown over the past several decades, several quantitative approaches have been developed to measure mitochondrial morphology in a robust and unbiased way. In developing Mito Hacker, we have benefited from many of these previously developed tools and approaches. Mito Hacker builds on these previous approaches and adds several new functionalities that significantly expand speed and utility. First, Cell Catcher provides a robust method to generate single cell images from multi-cell fluorescent images. This not only speeds the process for analysis for all experiments, but it enables single-cell, high throughput analysis of mitochondrial morphology that is not possible using manual identification of individual cells. While other tools exist to isolate single cells from multi-cell images, such as cell profiler, these tools are not specifically designed to function with mitochondrial stained images and do not have seamless integration with mitochondrial segmentation and analysis^40^. Second, Mito Hacker integrates the typical experimental workflow to allow a researcher to go from images to data set within the same platform. At the same time, while Cell Catcher, Mito Catcher and MiA will communicate with each other, each of the three tools can also be used in isolation if desired, providing maximum flexibility. Finally, the morphometric analysis provided within MiA collects data at the sub-mitochondrial, mitochondrial and cellular levels. While many available tools report data at one or two of these levels, MiA reports data from all three. Furthermore, it introduces several unique cell-level measurements that quantify the distribution of the mitochondrial network throughout the cell, a feature that has traditionally received less attention than other morphological features and yet likely contributes to how mitochondrial function impacts cellular physiology.

The ability of Mito Hacker to rapidly collect morphological data from thousands of individual cells enables a number of analytical approaches that are not possible with the amount of data collected using more traditional approaches. This includes data science approaches such as machine leaning and neural networks. To that end, our understanding of the regulation of mitochondrial dynamics remains incomplete, as does our appreciation for how changing mitochondrial shape and distribution affect cellular physiology. Data science approaches hold the promise to uncover new relationships between these processes without many of the biases inherent in the more hypothesis-driven approaches. As a proof of this concept, we sought to determine whether Mito Hacker-generated data could be used to successfully predict Drp1 genotype, as Drp1 is a known regulator of mitochondrial shape. Importantly, we were able to successfully build a model to distinguish not only Drp1^−/−^ from Drp^+/+^ and Drp1^+/−^ cells, which had obvious morphological differences, but also Drp1^+/−^ and Drp1^+/+^ cells from each other, which were much more difficult to distinguish by eye. Moreover, the relative contributions of different features to the tree-based model indicates that mean mitochondrial density and mean mitochondrial area, readouts that reflect mitochondrial width and size, were most useful in separating these three populations of cells. We learn several important things from this exercise: First, our ability to distinguish these three cell lines validates both the quality of the data generated by Mito Hacker and the idea that rich data sets of mitochondrial morphology enable prediction of Drp1 status, suggesting this approach can be leveraged to identify new regulators of mitochondrial shape; second, we learned that mitochondrial features predicted to be affected by changes in Drp1 activity (size) are driving the predictive model, validating that the model building exercise can generate novel biological insights; third, we learned that additional features not necessarily predicted to be affected by Drp1 (width) also contribute to the predictive model, suggesting the possibility of new insights into Drp1 function to be explored by these analyses.

Collectively, the results from our analysis and from the data modeling suggest that application of Mito Hacker and machine learning will be able to uncover novel biological insights when used in conjunction with other “omics” approaches on large panels of heterogeneous cell lines. For example, analysis of mitochondrial morphology on a large set of tumor cell lines isolated from diverse patient populations and with heterogeneous patterns of oncogene activation could lead to the identification of novel oncogenic pathways that regulate mitochondrial shape or the potential identification of relationships between mitochondrial morphology and patient outcome. Ultimately, these tools offer an unbiased and complementary approach to the more traditional approaches of biological inquiry and will serve to accelerate the pace of discovery.

## Methods

### Generation of tumor-derived cell lines

The generation of murine pancreatic tumor cell lines KPDC253, KPDC145 and KPDC143 was described previously^34^. Briefly, a ~ 10 mm^3^ tumor piece was isolated at necropsy from mice with genetically-induced pancreatic ductal adenocarcinoma. The tumor was mechanically and enzymatically digested with 2 mg/mL Collagenase (MP Biomedical) in DMEM supplemented with 10% FBS for 30 min at 37 °C. All tumor tissues were cultured overnight in DMEM supplemented with 10% FBS. Adherent cells were isolated, serially diluted, and plated in 96-well plates. Single cell clones were identified and expanded and the Drp1 genotype was confirmed by PCR. In accordance with institutional guidelines, all mice used in the generation of these cell lines were monitored and euthanized when they reached pre-determined endpoints or exhibited features associated with disease such as weight loss. All animal studies and procedures were approved by the University of Virginia Institutional Animal Care and Use Committee.

### Immunofluorescence

The described tumor-derived cell lines were plated on glass microslides the previous day, then transiently transfected with mitochondria-targeted YFP (BD Biosciences) and incubated for 24 h. Cells were fixed with 4% formaldehyde (Cell Signaling Technologies) and mounted immediately in Prolong Gold antifade reagent with DAPI (Life Technologies). Alternatively, mitochondria can be stained with 100 nM MitoTracker Red CMXRos (Life Technologies) for 45 min prior to fixation. Note that Mito Hacker can also be used to analyze cells that have been immuno-stained for mitochondrial proteins after fixation and nuclei can alternatively be visualized with a Hoechst stain. A Zeiss LSM 710 confocal microscope with 63X oil objective was used for imaging. Single z-slice images were acquired at 8-bit depth and 1024 × 1024 or 2048 × 2048 size.

### Using Mito Hacker

Mito Hacker is a collection of independent tools aimed to process cellular mitochondrial images at different levels of specificity; 2d Multi-cell RGB images, 2d single-cell RGB images, and 2d binary single-cell images. Mito Hacker is developed in python 3.7 and uses 14 different python libraries across different tools to analyze the images. All the tools offer interactive, semi graphical user interface through Jupyter notebooks and ipywidgets. Installation of Anaconda distribution of python will satisfy all the requirements of Mito Hacker, except OpenCV package which can be independently installed after installing Anaconda. The instructions to download and install all the Mito Hacker requirements can be found at Mito Hacker GitHub repository (https://github.com/Mitogenie/Mito-Hacker).

Once all the requirements are satisfied, the users can either download or clone the repository on their computers. Mito Hacker runs on Linux, Mac, and Windows operating systems without any modifications. Further information about Mito Hacker project, including the list of libraries, project folder structure, and different tools Input/output guide is available on the GitHub repository readme page.

After downloading the Mito Hacker directory, based on the image type, the user can start the analysis from any of the tools (Supplementary Fig. 11). Each tool follows a series of logical guided steps to process the data. When user interaction is required, the user is prompted to take appropriate action.

#### Cell Catcher

This tool accepts multi-cell RGB images, and outputs single-cell RGB images. First, the user locates the desired files on the computer by entering the address of the files. The next step is to randomly sample some images that are used to tune the model parameters. The user sets the desired number of sample images. Model tuning has two steps. The first step is tuning the parameters of the nuclei segmentation model. Once set, the next step is to set a global mitochondrial threshold for cell-separation purposes. The user can use different settings and experiment by separating the sample images to find the set of parameters that best isolates the single cells from the sample images. Once the model parameters are set, the final step is to analyze all of the images using the tuned parameters. The output of Cell Catcher is a set of single-cell RGB images that are separated from the input images. These images can be used by Mito Catcher to segment their mitochondrial network, or they can be used for other purposes, such as training ML algorithms.

#### Mito Catcher

This tool accepts single-cell RGB images, and outputs single-cell binary images. The input images can either come from Cell Catcher, or can be provided by the user. Similar to Cell Catcher, the process starts with locating and sampling input images. Next, the user selects a filter to segment the input data. Once the filter is selected, the next step is to fine-tune the parameters of the selected filter to find a combination of parameters that results in the most reliable segmentation of the mitochondrial network. The last step is to analyze all the images using the selected filter and its set of tuned parameters. The output of Mito Catcher is a set of single-cell binary images. These images can either be used by MiA for quantifying the mitochondrial network of the cells, or they can be used by the user for other purposes, like training external ML models.

#### MiA

This tool accepts single-cell binary images, and outputs two CSV files containing raw and processed measurements on the input cells. Like the other two tools, the process starts by locating the desired images on the computer. Next, the user chooses a desired name for the output files, and the last step is bulk analysis of the images.

### Modeling

For generating predictive models, we imported the data using Pandas, and prepared them for modeling using the Sklearn library. All the models were created using Sklearn except the XGBoost model which was created by XGBoost library. Cross validation, and model evaluation with different metrics were also performed using Sklearn. We tuned the hyper parameters for the XGBoost model through an exhaustive grid search with tenfold cross-validation. The feature importance and tree structure were generated by XGBoost and were plotted using Matplotlib, Seaborn, and Graphviz libraries.

## Supplementary information

Supplementary Information.

## Data Availability

The source code, the Jupyter notebook file (with a GUI), installation guide, and sample data are available online at https://github.com/Mitogenie/Mito-Hacker.
