# Supplementary material for: Mito Hacker: a set of tools to enable high-throughput analysis of mitochondrial network morphology
Source: Sci Rep. 2020 Nov 3;10:18941. doi: 10.1038/s41598-020-75899-5 (PMC7642274; doi:10.1038/s41598-020-75899-5)
Supplement: Supplementary file 1 — Supplementary Information. [file 41598_2020_75899_MOESM1_ESM.pdf]

Supplementary Information for:

Mito Hacker: A Set of Tools to Enable High-Throughput Analysis of Mitochondrial Network Morphology

Ali Rohani[1], Jennifer A. Kashatus[1], Dane T. Sessions[1], Salma Sharmin[1], David F. Kashatus[1,*]

[1]Department of Microbiology, Immunology and Cancer Biology, University of Virginia School of Medicine, Charlottesville, VA, 22908, USA

*kashatus@virginia.edu

Supplementary Figure 1

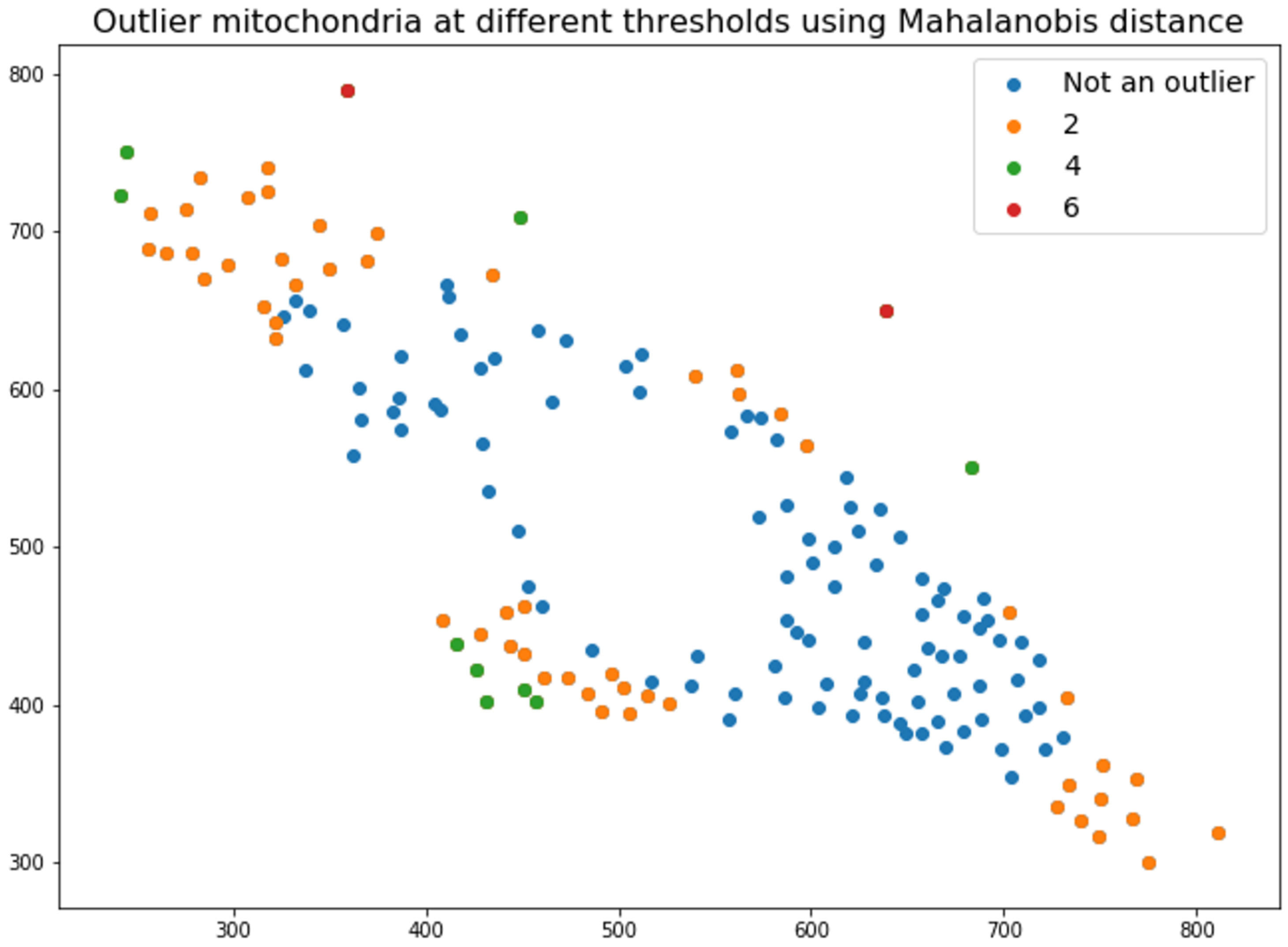

Supplementary Figure 1 - Two dimensional distribution of the centers of mitochondria across a cell. Colors represent the threshold level at which a given mitochondrion will be considered an outlier: Red: Threshold 6 (p=0.05), Green: Threshold 6 (p=0.135), Orange: Threshold 2 (p=0.37), Blue: Not an outlier. The p-values at each threshold indicate the chance of committing a type-I error in marking a mitochondrion as an outlier.

Supplementary Figure 2

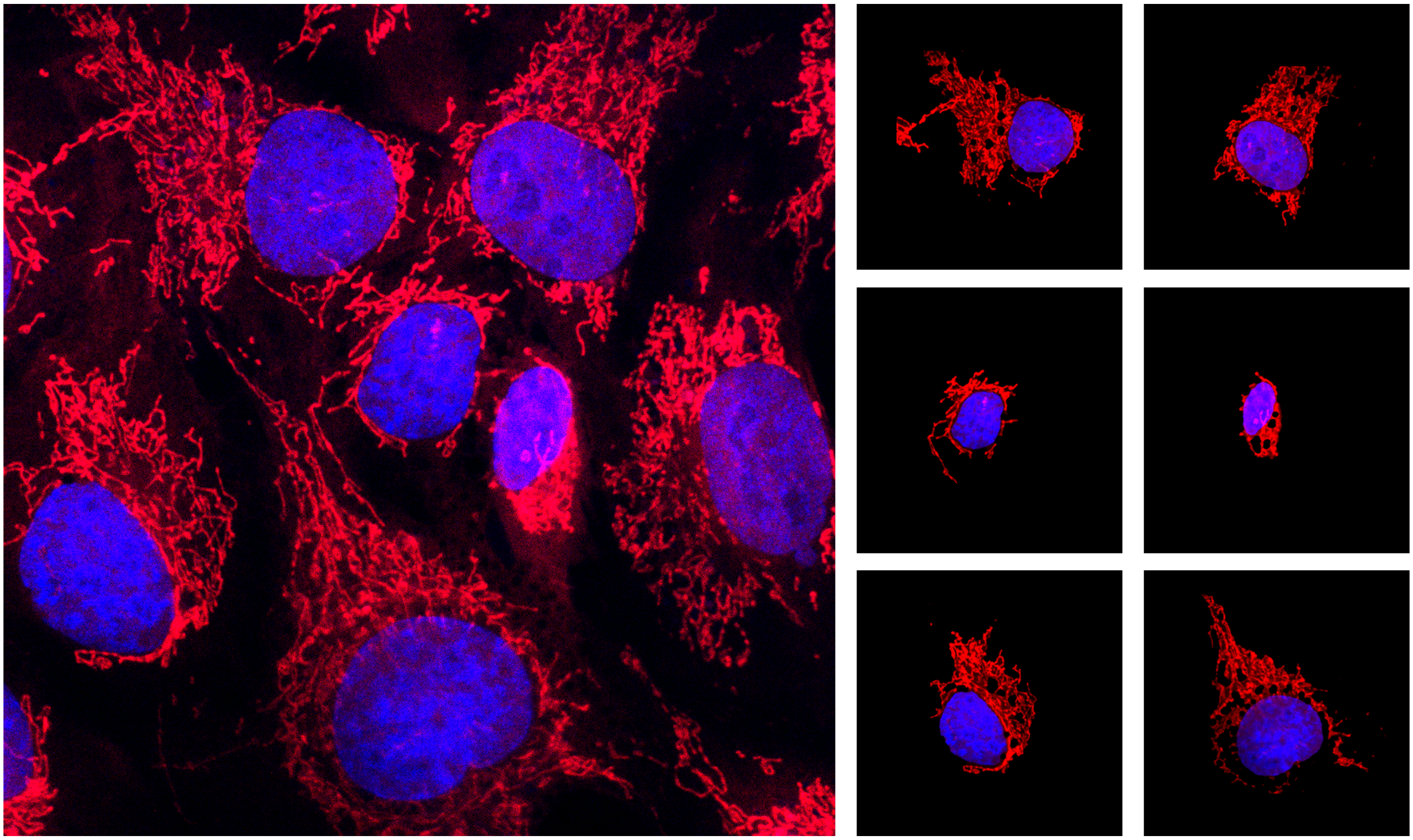

Supplementary Figure 2 - Isolation of single cell images of MitoTracker Red-stained HEK cells. Multi-cell confocal image was captured by staining mitochondria with Mitotracker Red CMXRos and nuclei with DAPI. Single-cell images were generated using Cell Catcher.

Supplementary Figure 3

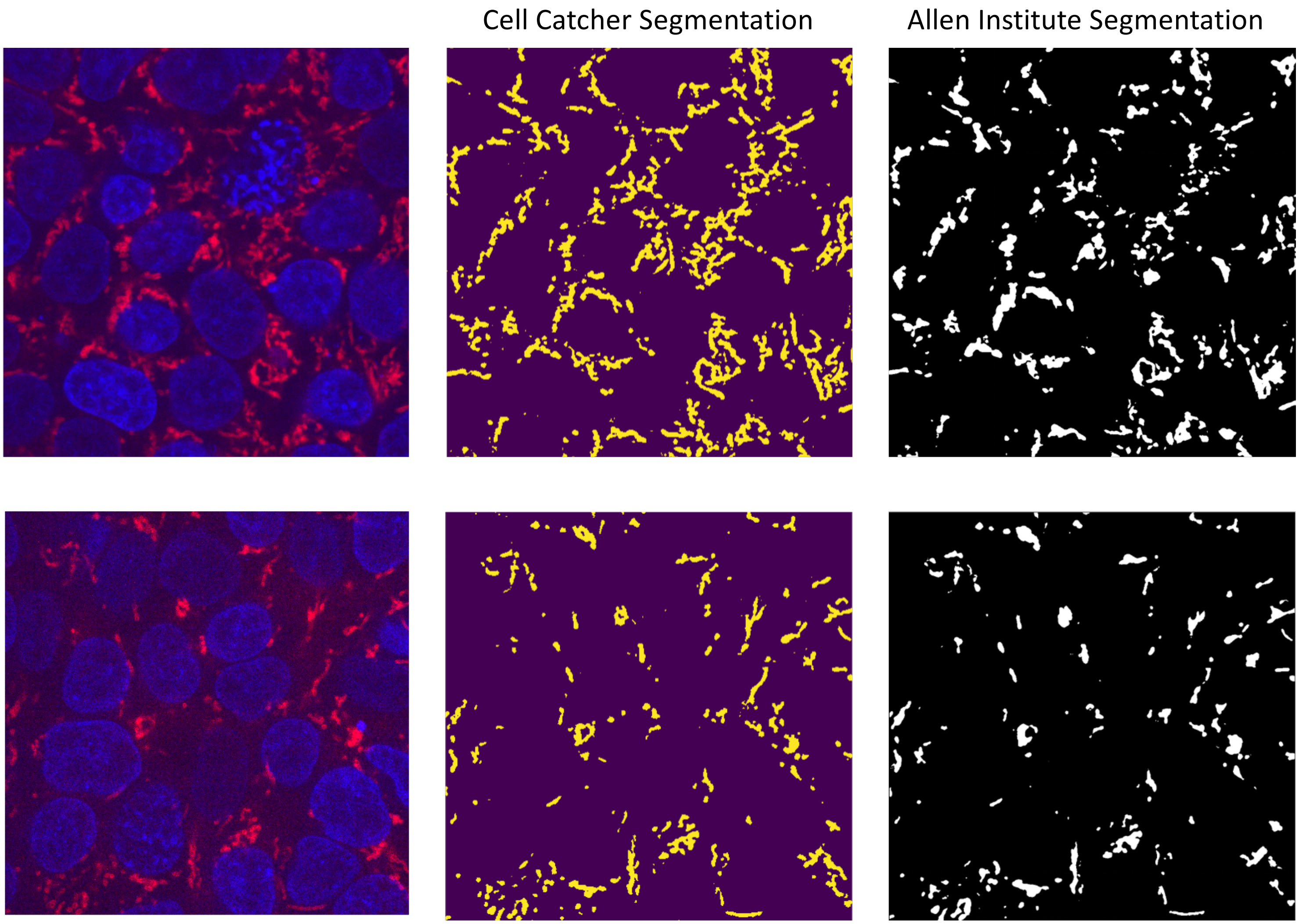

Supplementary Figure 3 - Performance of Cell Catcher on publicly available data set. Mitochondrial segmentation from the Allen Institute's Cell Feature Explorer (AICS). Initial mitochondrial segmentation in Cell Catcher identifies potential mitochondrial objects and assigns them to different cells for isolating and separating them from multi-cell images. Images on the right represent the mitochondrial segmentation provided with the original images.

Image Source: https://cfe.allencell.org/

Supplementary Figure 4

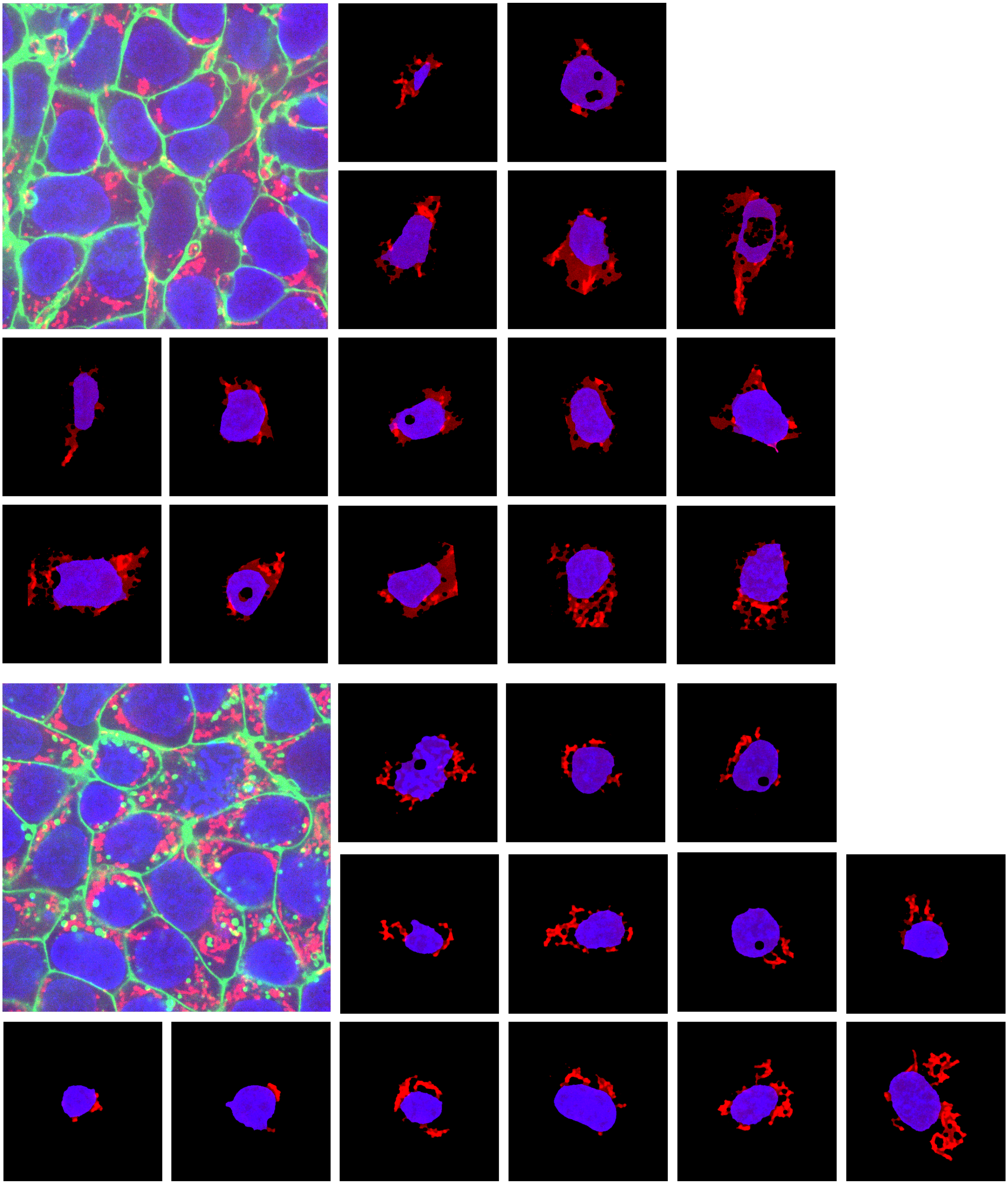

Supplementary Figure 4 - Performance of Cell Catcher on publicly available data set. Isolation of individual cells from the Allen Institute's Cell Feature Explorer (AICS). Cell separation was performed on images lacking the plasma membrane channel (green). The cells touching the image frame are automatically discarded by Cell Catcher.

Image Source: https://cfe.allencell.org/

Supplementary Figure 5

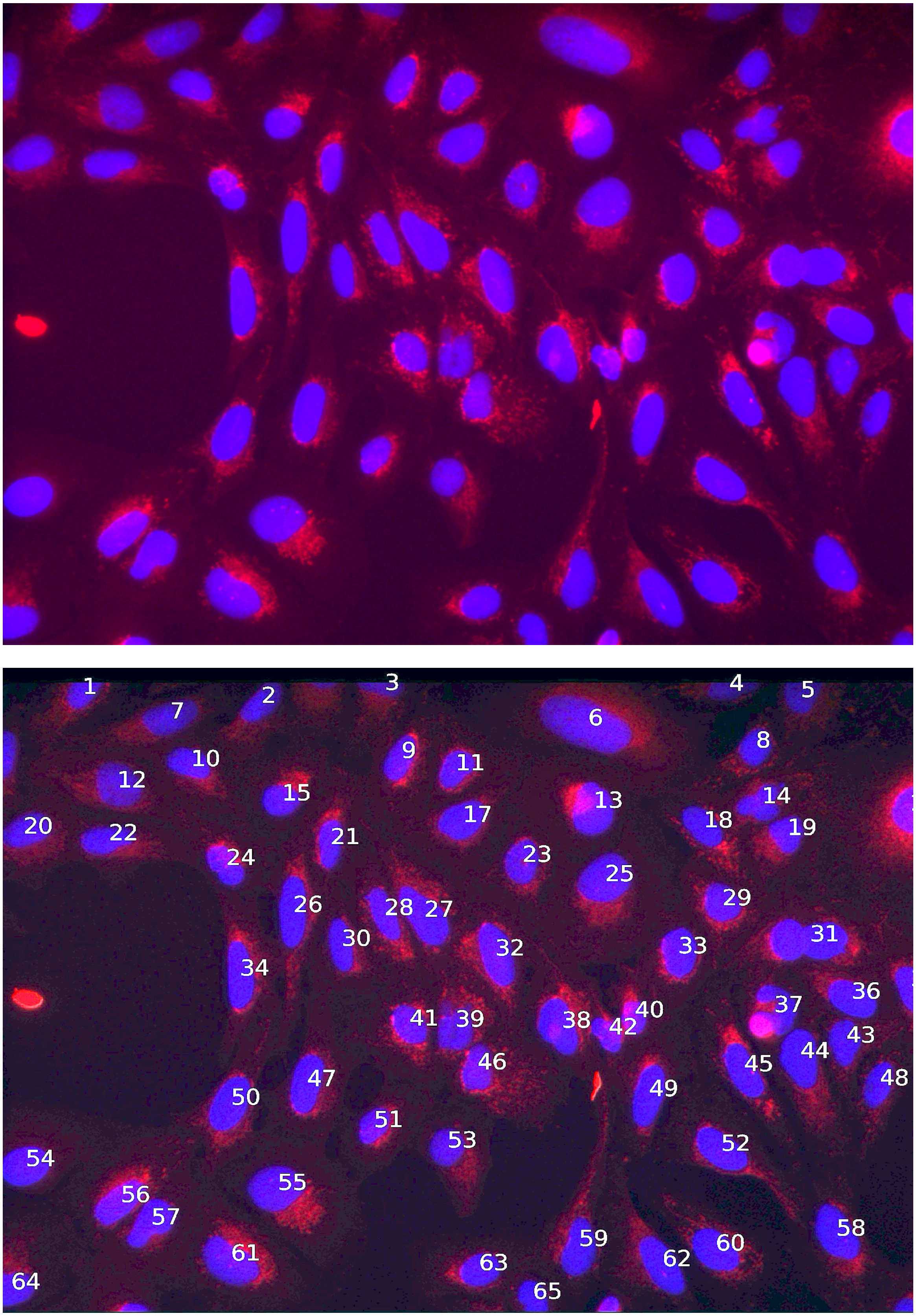

Supplementary Figure 5 – Performance of Cell Catcher on publicly available data set. Cell Catcher was able to successfully identify and isolate cells from the following public dataset: Human U2OS cells – compound-profiling Cell Painting experiment. The sample image, with 65 cells, had a low SNR and relatively low resolution (696*520).

Image source: https://data.broadinstitute.org/bbbc/BBBC022/

Supplementary Figure 6

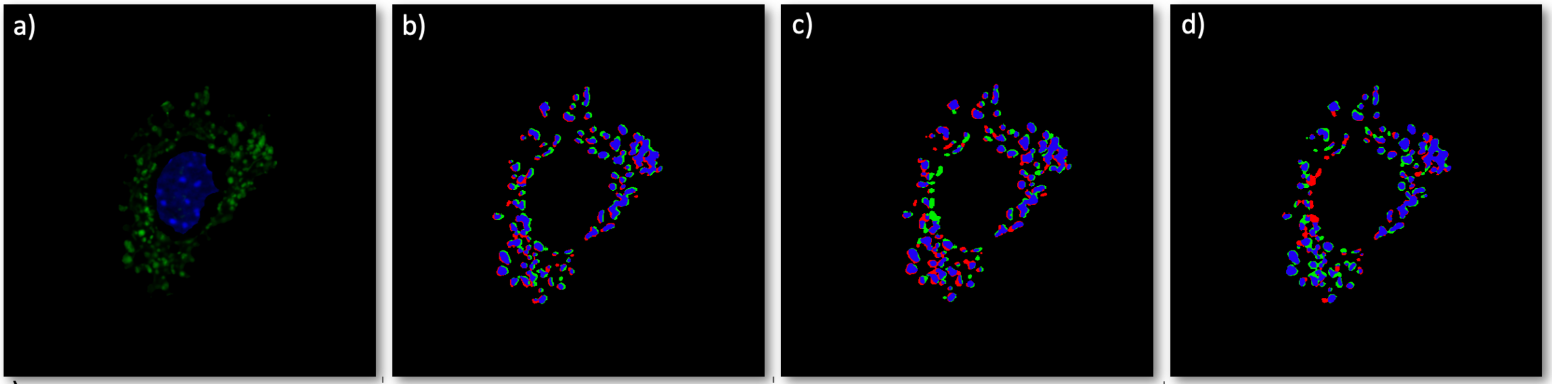

e)

Effective overlap	Range: (47% : 76%)
	Average: 62%
% of reference user markings covered by Mito Catcher markings	Range: (65% : 97%)
	Average: 76%
ratio of mitochondrial content marked by Mito Catcher versus the reference user	Range: (0.96 : 1.20)
	Average: 1.06
% of effective overlap change by using AdaMM algorithm (optimum settings)	Range: (-0.1% : 2.2%)
	Average: 1.4%
Average performance change at different tile sizes for test images	16: -2.6%
	32: -0.8%
	64: 1.4%
	128: -0.4%
	256: 0.0%

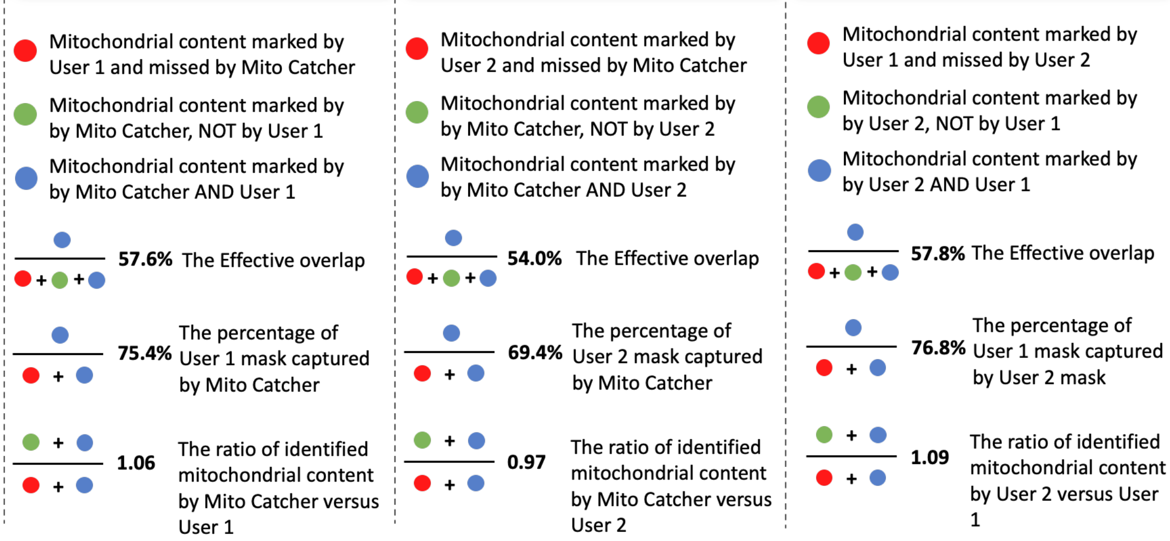

Supplementary Figure 6 – Comparison of mitochondrial network segmentation by Mito Catcher and manual segmentation demonstrates segmentation performance by Mito Catcher is comparable to human. a) A sample cell that was segmented by Mito Catcher and two different users. Comparison of segmented mitochondria b) Mito Miner vs. user1. c) Mito Miner vs. user2 d) user1 vs. user2. e) Summary of Mito Miner performance with different settings.

Supplementary Figure 7

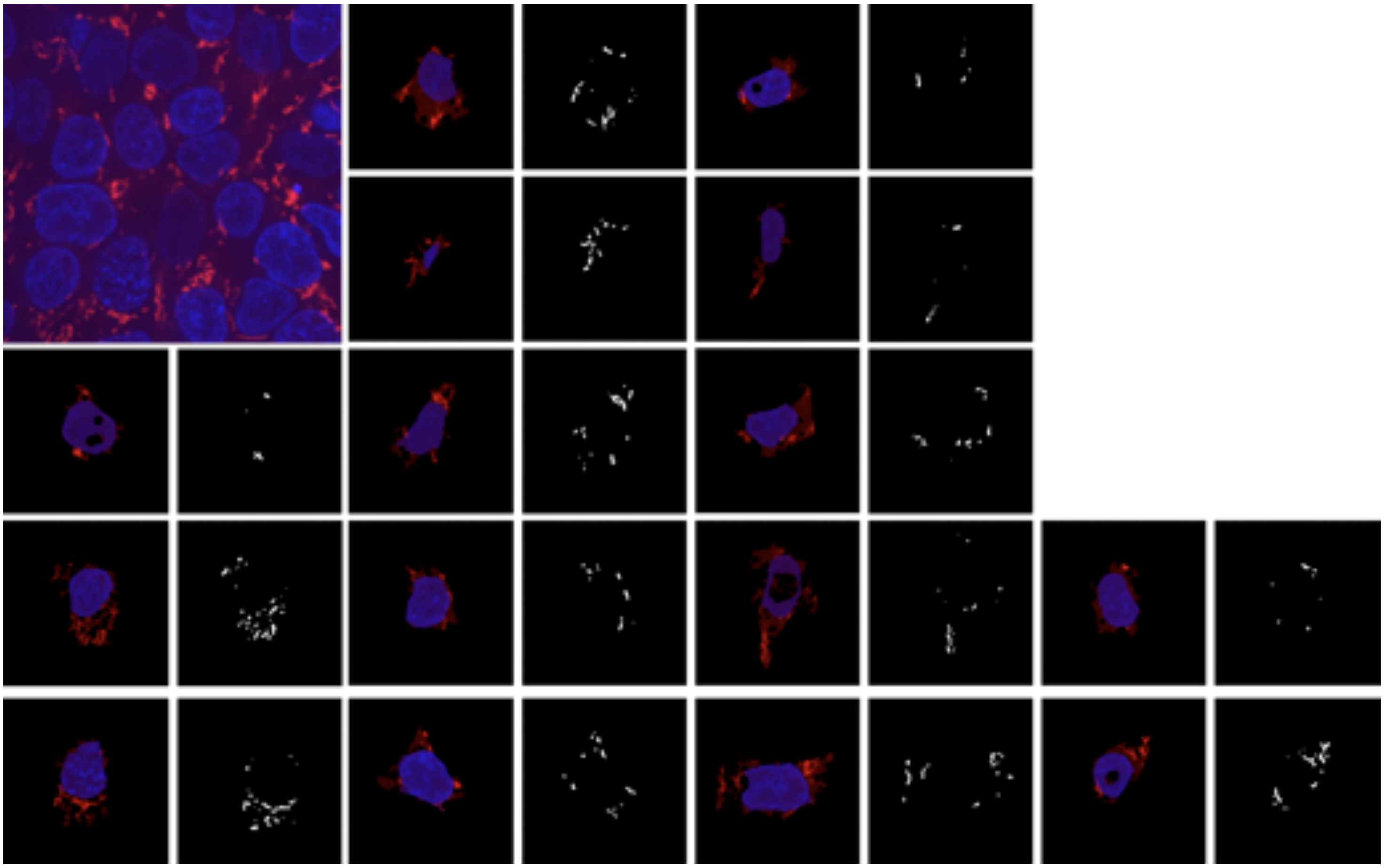

Supplementary Figure 7 - Performance of Mito Miner on publicly available data set. Mitochondrial segmentation on individual images of cells from the Allen Institute's Cell Feature Explorer (AICS) generated by Cell Catcher.

Image Source: https://cfe.allencell.org/

Supplementary Figure 8

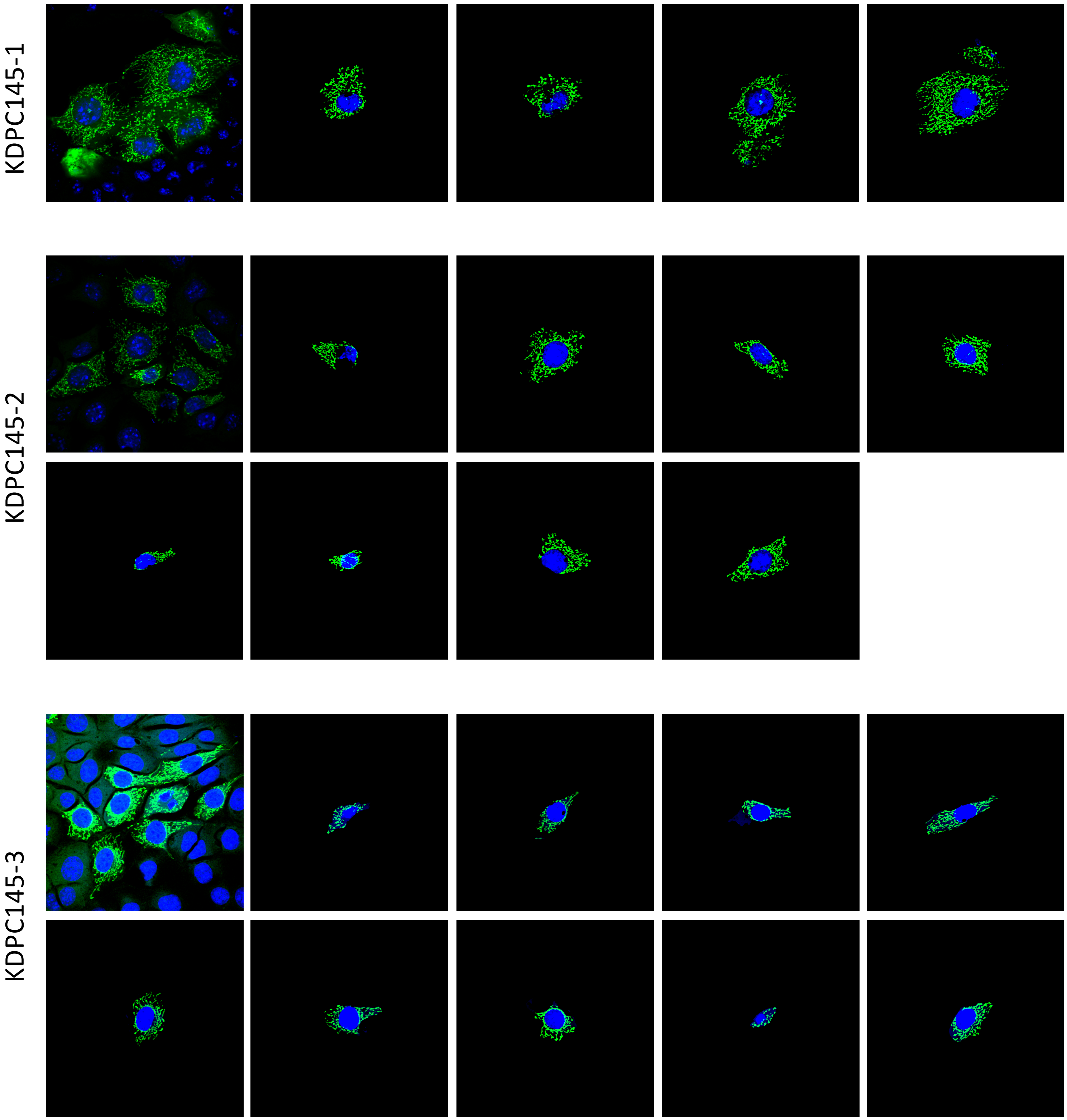

Supplementary Figure 8 – Isolation of single cell images of KPDC145. Multi-cell images of tumor cell line KPDC145 were captured following transfection with mito-YFP. Single-cell images were obtained using Cell Catcher.

Supplementary Figure 9

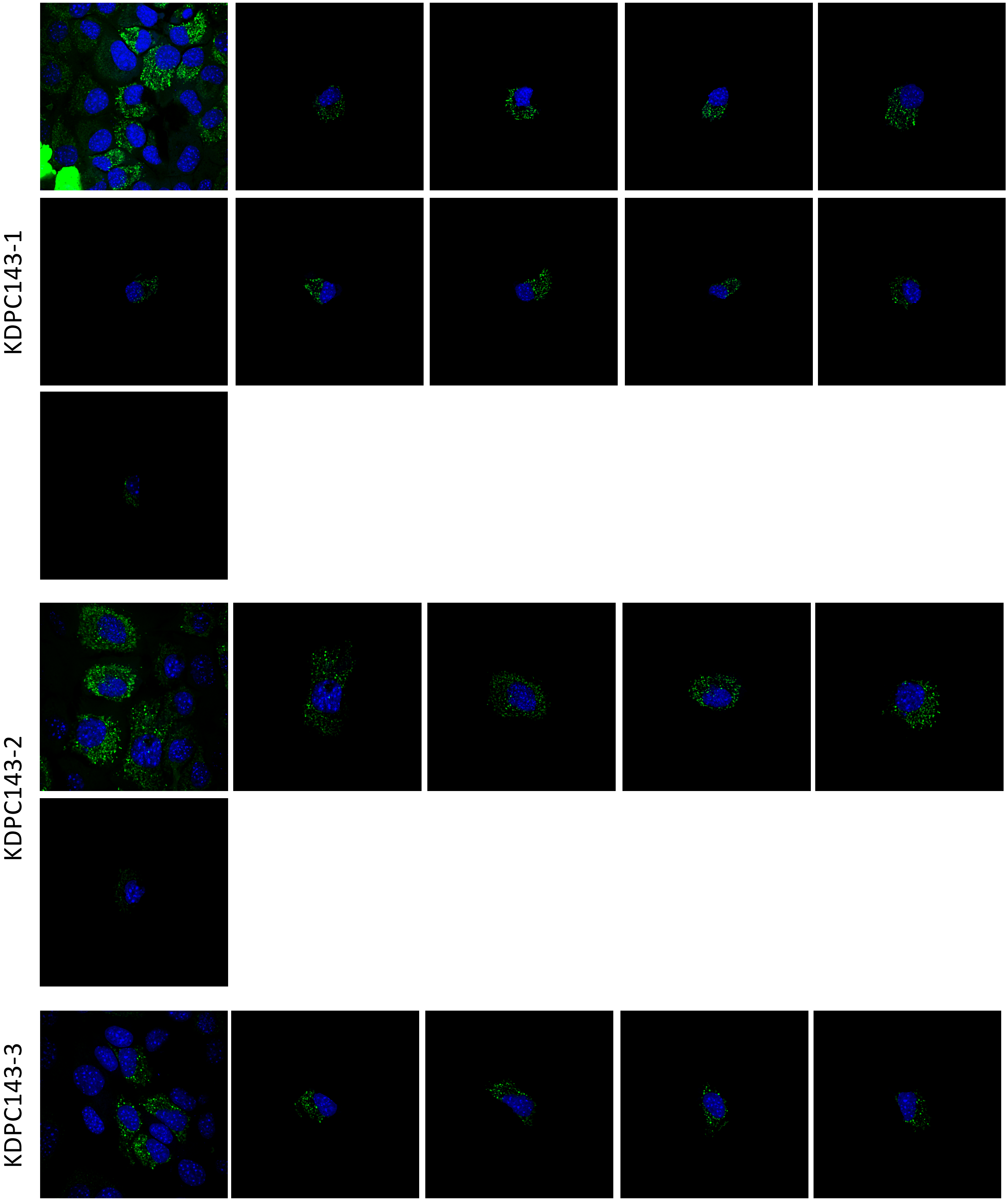

Supplementary Figure 9 – Isolation of single cell images of KPDC143. Multi-cell images of tumor cell line KPDC143 were captured following transfection with mito-YFP. Single-cell images were obtained using Cell Catcher.

Supplementary Figure 10

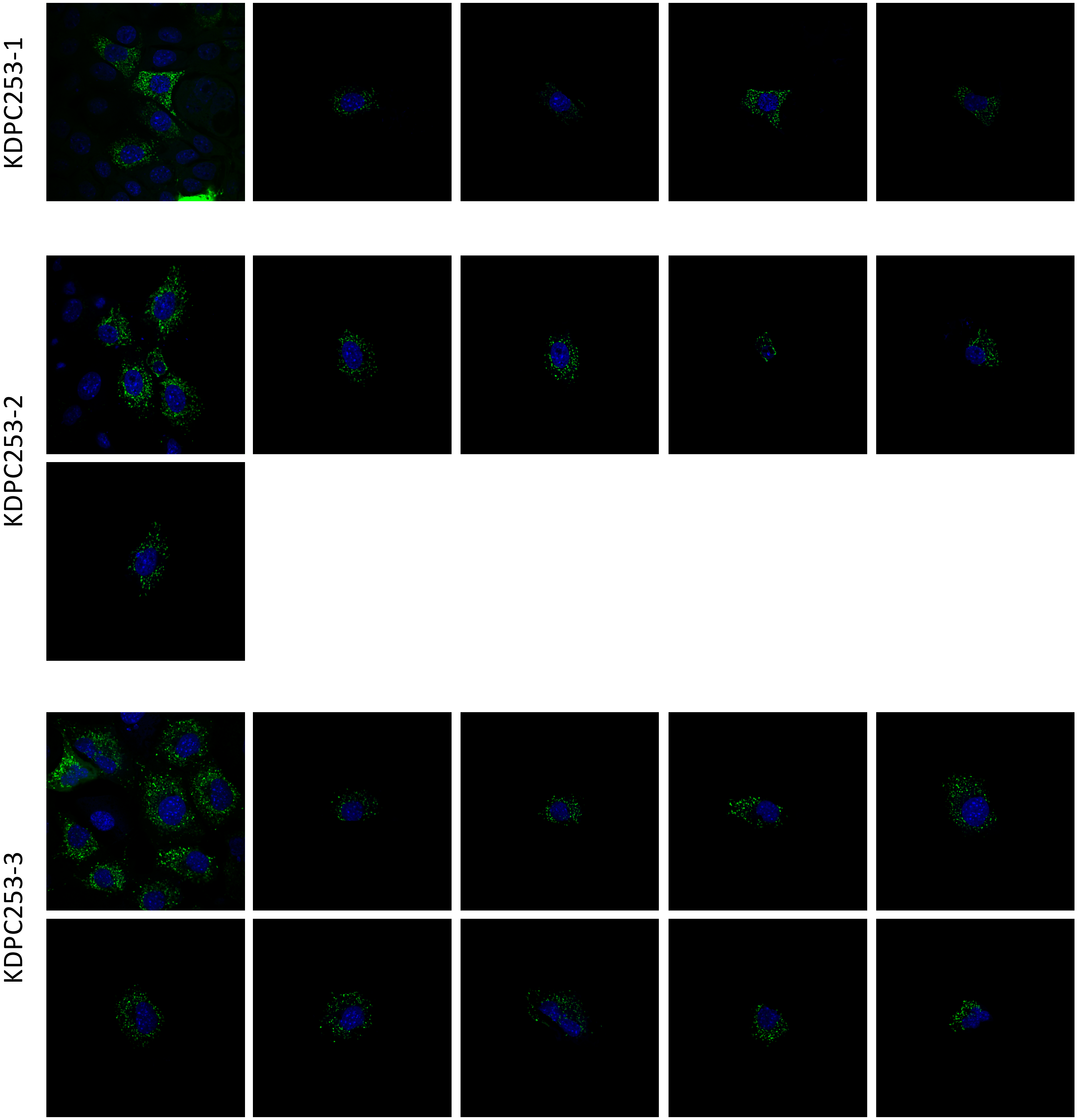

Supplementary Figure 10 – Isolation of single cell images of KPDC253. Multi-cell images of tumor cell line KPDC253 were captured following transfection with mito-YFP. Single-cell images were obtained using Cell Catcher.

Supplementary Figure 11

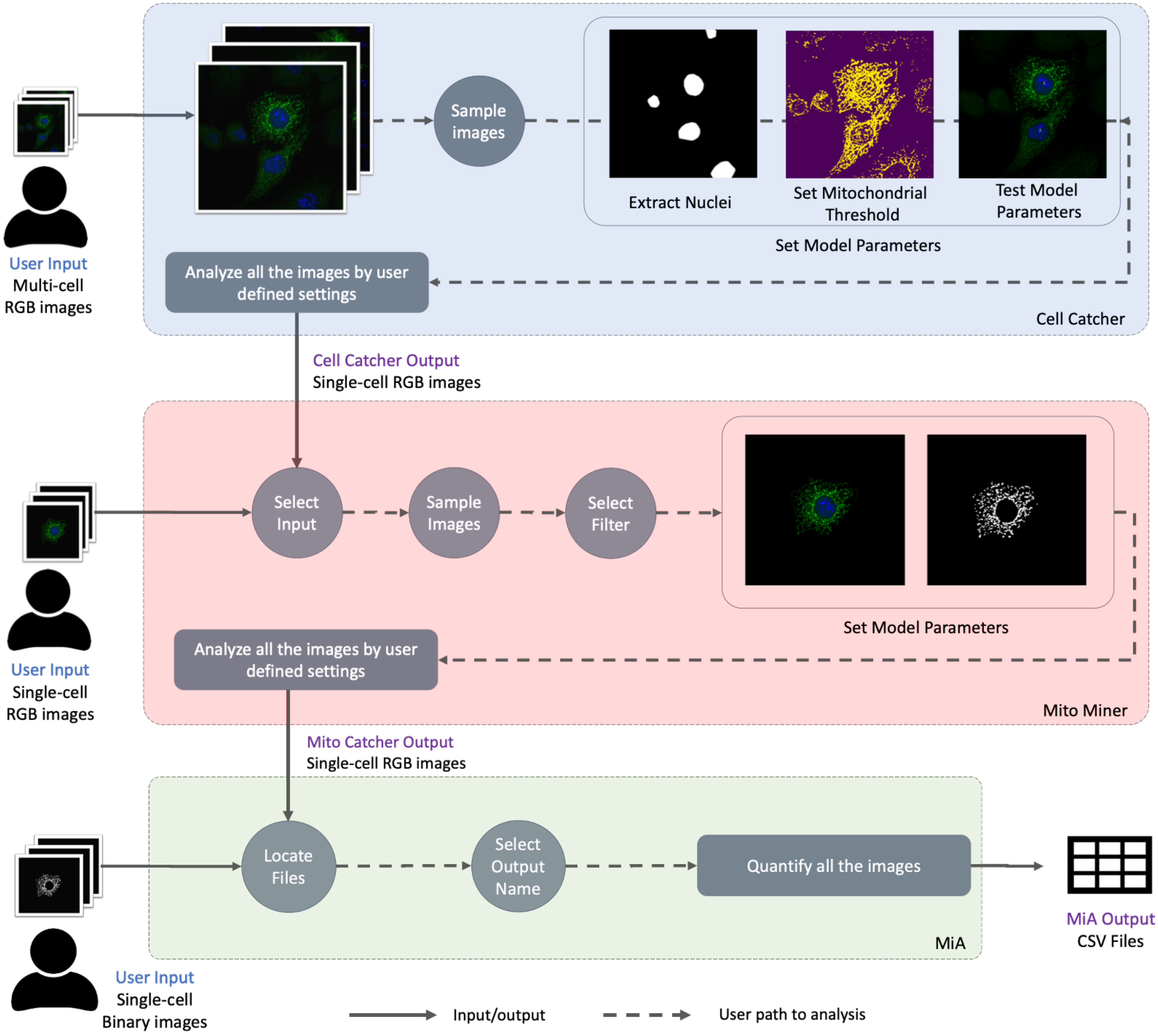

Supplementary Figure 11 – Mito Hacker workflow. Based on the type of image available, the user can initiate analysis from multiple different starting points. The tools can be used in the following series: Cell Catcher > Mito Catcher > MiA, or each can be used independently

Table 1: Description of Mito Hacker Features

Mitochondria Level Measurements	
mito_area	Area of individual mitochondrion.
mito_centroid	Position of individual mitochondrion.
mito_eccentricity	Eccentricity of the ellipse with the same second-moments as the mitochondrion, and it is defined the ratio of the focal distance of the ellipse over the major axis length.
mito_equi_diameter	The diameter of a circle with the same area as the mitochondrion.
mito_euler_number	Euler characteristic of the mitochondrion. Computed as 1-number of holes in the mitochondrion.
mito_extent	Ratio of the area of the mitochondrion to the area of its bounding box.
mito_major_axis	The length of the major axis of the ellipse that has the same normalized second moments as the mitochondrion.
mito_minor_axis	The length of the minor axis of the ellipse that has the same normalized second moments as the mitochondrion.
mito_orientation	Angle between the horizontal line and the major axis of the ellipse that has the same second moments as the mitochondrion, ranging from $-\pi/2$ to $\pi/2$ counter-clockwise.
mito_perimeter	Perimeter of the mitochondrion.
mito_solidity	Ratio of the area of each mitochondrion to the area of the convex hull surrounding the mitochondrion.
mito_centroid_x	Distance of the mitochondrion center from the center of the nucleus in the corresponding cell in the X direction.
mito_centroid_y	Distance of the mitochondrion center from the center of the nucleus in the corresponding cell in the Y direction.
mito_distance	Euclidian distance of the mitochondrion center from the center of the nucleus in the corresponding cell.
mito_weighted_cent_x	Distance of the mitochondrion center from the center of the nucleus in the corresponding cell in the X direction, weighted by mitochondrion area.
mito_weighted_cent_y	Distance of the mitochondrion center from the center of the nucleus in the corresponding cell in the Y direction, weighted by mitochondrion area.
mito_weighted_distance	Euclidian distance of the mitochondrion center from the center of the nucleus in the corresponding cell, weighted by mitochondrion area.
mito_form_factor	[(1/4π).(mito_perimeter^2/mito_area)]
mito_roundness	[4(mito_area)/(π.mito_major_axis^2)]
mito_branch_count	Number of branches in the skeleton of the mitochondrion

mito_total_branch_length	Total length of the branches in the skeleton of the mitochondrion.
mito_mean_branch_length	Average length of the branches in the skeleton of the mitochondrion.
mito_median_branch_length	Median length of the branches in the skeleton of the mitochondrion.
mito_std_branch_length	Standard deviation of the lengths of the branches in the skeleton of the mitochondrion.
mito_mean_branch_angle	Average angle of the branches in the skeleton of the mitochondrion.
mito_median_branch_angle	median angle of the branches in the skeleton of the mitochondrion.
mito_std_branch_angle	Standard deviation of the angles of the branches in the skeleton of the mitochondrion.
mito_total_density	Sum of the distances of the pixels in each mitochondrion from the boundary of the mitochondrion. This feature is an alternative method for measuring the size each mitochondrion.
mito_average_density	Average of the distances of the pixels in each mitochondrion from the boundary of the mitochondrion. This feature effectively measures the width of each mitochondrion.
mito_median_density	median of the distances of the pixels in each mitochondrion from the boundary of the mitochondrion. This feature effectively measures the width of the mitochondrion.
**Cell Level Measurements**<br>**Mitochondrial aggregate measurements**	
cell_mean_mito_FEATURE	Average of the FEATURE distribution from all the mitochondria in the cell.
cell_median_mito_FEATURE	Average of the FEATURE distribution from all the mitochondria in the cell
cell_std_mito_FEATURE	Standard deviation of the FEATURE distribution from all the mitochondria in the cell
**Cell level measurements based on mitochondria distribution**	
cell_kurtosis_x	Kurtosis of mitochondria distribution across the cell in X direction.
cell_weighted_kurtosis_x	Kurtosis of mitochondria distribution across the cell in X direction, with distances normalized by the mitochondrial area.
cell_kurtosis_y	Kurtosis of mitochondria distribution across the cell in Y direction.

cell_weighted_kurtosis_y	Kurtosis of mitochondria distribution across the cell in Y direction, with distances normalized by the mitochondrial area.
cell_kurtosis_squared	Kurtosis of mitochondria distribution across the cell
cell_weighted_kurtosis _squared	Kurtosis of mitochondria distribution across the cell, with distances normalized by the mitochondrial area
cell_skewness_x	Skewness of mitochondria distribution across the cell in X direction.
cell_weighted_skewness_x	Skewness of mitochondria distribution across the cell in X direction, with distances normalized by the mitochondrial area.
cell_skewness_y	Skewness of mitochondria distribution across the cell in Y direction
cell_weighted_skewness_y	Skewness of mitochondria distribution across the cell in Y direction, with distances normalized by the mitochondrial area
cell_skewness_squared	Skewness of mitochondria distribution across the cell
cell_weighted_skewness _squared	Skewness of mitochondria distribution across the cell, with distances normalized by the mitochondrial area.
cell_network_major_axis	The length of the major axis of the ellipse that has the same second moments as the convex hull from the distribution of the mitochondria across the cell (as a simple estimation of cytoplasm)
cell_network_minor_axis	The length of the minor axis of the ellipse that has the same second moments as the convex hull from the distribution of the mitochondria across the cell (as a simple estimation of cytoplasm)
cell_network_orientation	Angle between the horizontal line and the major axis of the ellipse that has the same second moments as the convex hull from the distribution of the mitochondria across the cell (as a simple estimation of cytoplasm), ranging from $\pi/2$ to $\pi/2$ counter-clockwise.
cell_network_eccentricity	Eccentricity of the ellipse with the same second-moments as the convex hull from the distribution of the mitochondria across the cell, and it is defined the ratio of the focal distance of the ellipse over the major axis length.
cell_network_effective _solidity	Total mitochondria area in the cell, divided by the area of the convex hull from the distribution of the mitochondria across the cell.
cell_network_fractal _dimension	The fractal dimension of the mitochondrial network in the cell.

Aside I: Mito Hacker's various functions and their parameters

To ensure the best results, we highly suggest to analyze images from the same experiment in each batch or round of analysis. In this case, the images in each batch are more or less similar, and as a result, the processing on the images will result in more uniform and reliable results across the images in each batch.

Cell Catcher

Identifying nuclei in the image

As the name suggests, this function is used to identify and segment individual nuclei in the image. The nuclei play an important role in cell separation. They are used as the initial seed to detect and isolate individual cells. To deal with a large range of images with different levels of signal, noise, intensity, and nuclei sizes this function employs a set of tasks to process the images. While the default parameters are highly tuned to immediately work with a wide range of images, there is not a set of parameters that works for all. The users should find a combination of parameters that best represent the nuclei in their images, though they should maintain consistent parameters across a given experimental set. Below is the list of parameters for this function:

Nuclei Signal Threshold

- By changing the value of this parameter, you change the signal threshold for detecting nuclei in the image. Lower threshold may help you to detect dimmer nuclei in the image. However, in some cases extremely low threshold values may cause problems by capturing the background noise as nuclei.

- Select a threshold value that reasonably works for your sample images. You may check sample images by selecting them from the dropdown menu.

Nuclei Non-Uniformity

- Different nuclei in the same image may have different signal intensity and signal distribution. This option helps the user to reach a better nuclei segmentation results over a wider range of images with varying nuclei across the image.

Nuclei Size Threshold

- This option helps the user to set the minimum acceptable nucleus size in the images, and reduce noise.

Correct Nuclei Shape

- This feature tries to reconstruct the shape of the poorly illuminated nuclei in the image.

- In some cases, this correction may result in nuclei with angular shapes.

  - This phenomenon usually does not have an adverse effect on the analysis, since these binary nuclei are merely used as masks on the real nuclei in the image, and their goal is maximal capturing of the nuclei in the image. Ultimately the original shape of the nuclei (from the original image) would be presented in the image.

Low Nuclei Signal

- In cases where the signal level in the nuclei channel of your images is low, or the images suffer from low contrast, this option may help user to maximize nuclei capturing in the image.

Identifying mitochondria in the image

This function aims to identify mitochondria on the image and assign them to different cells across the image to separate individual cells. Cells on across set of images have a wide range of mitochondrial network distribution, signal and noise intensities. This function performs multiple processing steps on the images to ensure maximal mitochondrial capturing. We have tuned the default parameters to work with a wide range images, but again there is not set of parameters that works for all. The users should find a combination of parameters that best represents the mitochondrial distribution in their images, to ensure all mitochondria are captured and assigned to different cells, while the noise is cut. Below is the list of parameters for this function:

Mitochondrial Signal Threshold

- This parameter sets the intensity threshold for mitochondria. Lower threshold results in capturing more possible mitochondria in the image, but has the potential to capture excessive noise. This is further explained later in the text under "Global Mitochondrial Mask" section.

Mitochondrial Search Radius for Ghost Cells

- This parameter sets the radial distance around the nuclei to search for mitochondria. This tool is used to identify the ghost cells in the image.
  - As discussed in the main text, the ghost cells are the cells where their nucleus is stained but mitochondrial staining is missing.

Maximum Mitochondrial Content for Ghost Cells

- This parameter sets the minimum acceptable amount of mitochondrial content around a cell (within the radius set above). The cells with mitochondrial content below this threshold will be marked as ghost cells.

Remove the Ghost Cells

- By selecting this option, the function removes the nuclei of the cells marked as ghost cells from the image.

Low Mitochondrial Signal

- You may select this option if the signal level, or the contrast (Signal to Noise Ratio (SNR)) in the mitochondrial channel is low in the set of images you analyze.
    - Based on the image condition this option may have additional applications. That we will discuss in more details in the "Important Notes" section.

My Cells Are Reasonably Separated

- If your cells are sparsely distributed across your images, you can use this option along with "Low Mitochondrial Signal" option to speed up your cell separation up to 10X. Please refer to the "Important Notes" section.

Separate Cells

- This box should be checked in order to separate the individual cells in the image.
    - Please refer to the additional notes below to find the best time to check this box.

Important Notes

What does the "Global Mitochondrial Mask" figure tell you?

This function generates an image titled "Global Mitochondrial Mask" which serves an important purpose, and can be extremely useful if used and interpreted properly. This figure represents the global mitochondrial content mask for the image, and is not intended to reflect the final and detailed mitochondrial mapping or network in your cells (That's Mito Catcher's job). The yellow objects on the image reflect all the objects on the image that are assessed as the possible mitochondrial content in the image, and would be assigned to different nuclei. This may also include some background noise, which is okay, since Mito Catcher will take care of it.

Lowering the "Mitochondrial Signal Threshold" would result in increase in the number and the area of the yellow objects in the image. This means that you have more mitochondrial candidate objects, which may increase the chance of capturing of true mitochondria in each image.

- This is true as long as the yellow objects across the image do not overlap and/or you are not capturing too much noise instead of mitochondria (i.e. parts of the image that you are sure that are noise and not real mitochondria)
- More yellow content in the image means more assignment tasks, which in turn may increase the processing time.

- Important: check a few images (using the drop-down menu above) before you decide on your final threshold value, and make sure your set of parameters reasonably represents the mitochondrial content in all of those images, since the same threshold will be applied to the batch of images you are analyzing together.

- There is an exception which is discussed in the "My Cells Are Entangled" section.

When to use "Low Mitochondrial Signal"

- The most obvious situation is when you have low signal or low contrast images. Low signal levels, and low Signal to Noise Ratio (SNR), make it harder for Cell Catcher to detect mitochondria in the image and subsequently assign them to different cells. By selecting this option Cell Catcher will perform additional pre-processing on the images in attempt to capture more mitochondria.

- If you select this option in high signal/SNR images, it will result in forming of yellow clusters in the "Global Mitochondrial Mask" figure, which is totally fine if the following two conditions are met:

  - As a result of selecting this option you are not capturing excessive amounts of noise as mitochondrial candidates in the image (Similar to lowering the "Mitochondrial Signal Threshold").

  - The yellow blobs formed around cells, or in general the yellow objects across the image are not overlapping or excessively touching each other.

  - In both cases, this option may increase the processing time since Cell Catcher should assign more content to various cells. *

*There is an exception which is discussed in the "My Cells Are Entangled" part.

When to use "My Cells Are Entangled"

If your cells have mitochondrial networks that are entangled, and it is very hard to decide on the boundaries of the adjacent cells, you may use this option. However, this option may increase the processing time up to 10X.

- When you have cells with entangled mitochondrial networks, if you select "Low Mitochondrial Signal" and/or you drastically lower the "Mitochondrial Signal Threshold" value, your actions may result in overlap or excessive touching of the mitochondrial networks from adjacent cells that may make the processing and separation of cells harder.

Before selecting this option, you should make sure that this approach is appropriate for all the majority of the images in your batch.

When to check the "Separate cells" box?

In short, this is the last option you may select in this function.

- While you are deciding on the best set of parameters for your sample images (which can individually be selected from the dropdown menu at the top), we suggest to keep the "Separate Cells" unchecked. Once you are happy with the combination of your parameters, then select this box to isolate individual cells in your desired images.

- Every time you select a new cell, while the segment cell is checked, it will automatically start to segment the cells in the image, which may take some time. Once segmentation is done, the segmented cells will show up.

Mito Catcher

Mitochondrial Segmentation Function

Filter Type

There are two filters available in this function. Their underlying mechanism is very similar, yet they have been tuned differently to work with different sets of images, with different signal distributions. You may find one more suited to your images. You may try both of filters with default values as a starting point to choose a proper filter to further tune for your images.

Let It Go

- This filter is a better option for images with low background intensity, and generally works better with wider range of images.

Not One of Us

- Similar to the other filter, this filter also separates signal from noise based on the distribution of background intensity in the nucleus area. Generally, this filter may work better in case of strong background noise.

Filter Options

Dark Image

- This option only visually enhances the dark images and has no effect on the analysis. Selecting this option makes images 3X brighter, and it is useful for comparing images when signal level is low, and it is hard to visualize the original cells.

Filter Strength

- Determines the strength of the background removal filter.

Filter Harshness

- Determines if the background removal is harshly applied to a small group of images, or whether it is more softly applied to a wider range of images.

Adaptive Options

The settings of the adaptive option are effective only if the adaptive option is selected.

Adaptive Filter Tile Size

- The size of the tile over which the adaptive function samples the data and compares it with the reference intensity.

Adaptive Filter Power

- This option sets the strength of the adaptive function in correcting the local signal threshold.

Adaptive Filter Footprint

- Determines whether the correction factor should affect a wide, or short range of cells with different background intensities.

Remove Debris

- In certain conditions, the applied filters may result in artifacts in the image that may be interpreted as mitochondria. This option attempts to clean up and remove the noisy objects from the image.
